# Supplementary material for: Risk of metabolic disorders in childless men: a population-based cohort study
Source: BMJ Open. 2018 Aug 17;8(8):e020293. doi: 10.1136/bmjopen-2017-020293 (PMC6104745; doi:10.1136/bmjopen-2017-020293)
Supplement: Supplementary file 1 [file bmjopen-2017-020293supp001.pdf]

Supplementary table I: Fourteen different sources of data were used to identify diabetic cases among men included in MDC-CC.

**Source of data**	**Diabetes criteria**
The Swedish National Diabetes Register (NDR)	All registered individuals
The Diabetes 2000 Registry	All registered individuals
The HbA1c register at Clinical Chemistry, Malmö	Individuals with at least two HbA1c ≥ 6% (not on the same day)
The Swedish Hospital Discharge Register (also The National Inpatient Register (IPR))	Individuals with the ICD10 codes E10-E14 and O244-O249 (corresponding ICD7-9 codes)
The Swedish National Patient Register – Outpatient Care	Individuals with the ICD10 codes E10-E14 and O244-O249 (corresponding ICD7-9 codes)
The Swedish Cause-of-death Register	Individuals with the ICD10 codes E10-E14 and O244-O249 (corresponding ICD7-9 codes)
The Swedish Prescribed Drug Register	Individuals with ATC code A10
MPP baseline screening (1974-92)	Based on questionnaire, fB-glucose ≥ 6.5 mmol/L and glucose ≥ 11 mmol/L at 120 minutes OGTT
MPP 6-year rescreening (1981-89)	Based on questionnaire, fB-glucose ≥ 6.5 mmol/L and glucose ≥ 11 mmol/L at 120 minutes OGTT
MPP rescreening (2002-06)	Based on questionnaire, list of antidiabetic drugs and fP-glucose ≥ 7 mmol/L
MDC baseline screening (1991-96)	Based on questionnaire and list of antidiabetic drugs
MDC cardiovascular cohort baseline screening (1992-94)	Based on fB-glucose ≥ 6.5 mmol/L
MDC 5-year rescreening (1997-2001)	Based on questionnaire and list of antidiabetic drugs

MDC cardiovascular cohort rescreening (2007-12)	Based on questionnaire, list of antidiabetic drugs, fB-glucose ≥ 6.5 mmol/L verified by fP-glucose ≥ 7 mmol/L and glucose ≥ 11 mmol/L at 120 minutes OGTT
