# Supplementary material for: Risk of metabolic disorders in childless men: a population-based cohort study
Source: BMJ Open. 2018 Aug 17;8(8):e020293. doi: 10.1136/bmjopen-2017-020293 (PMC6104745; doi:10.1136/bmjopen-2017-020293)
Supplement: Author's manuscript [file bmjopen-2017-020293.draft_revisions.pdf]

BMJ Open

BMJ Open is committed to open peer review. As part of this commitment we make the peer review history of every article we publish publicly available.

When an article is published we post the peer reviewers' comments and the authors' responses online. We also post the versions of the paper that were used during peer review. These are the versions that the peer review comments apply to.

The versions of the paper that follow are the versions that were submitted during the peer review process. They are not the versions of record or the final published versions. They should not be cited or distributed as the published version of this manuscript.

BMJ Open is an open access journal and the full, final, typeset and author-corrected version of record of the manuscript is available on our site with no access controls, subscription charges or pay-per-view fees (http://bmjopen.bmj.com).

If you have any questions on BMJ Open's open peer review process please email info.bmjopen@bmj.com

BMJ Open

Risk of metabolic disorders in childless men - A population-based cohort study

Journal:	*BMJ Open*
Manuscript ID	bmjopen-2017-020293
Article Type:	Research
Date Submitted by the Author:	27-Oct-2017

Complete List of Authors:	bungum, ane; Bispebjerg Hospital, department of occupational and environmental health<br>Glazer, Clara; Bispebjerg Hospital, department of occupational and environmental health<br>Bonde, Jens Peter; Copenhagen University Hospital Bispebjerg, Department of Occupational and Environmental Medicine<br>Nilsson, Peter; University of Lund, Clinical Sciences<br>Giwercman, Aleksander; Lund University, Department of Translational Medicine<br>Søgaard Tøttenborg, Sandra; Bispebjerg Hospital, department of occupational and environmental health

**Primary Subject Heading**:	Reproductive medicine
Secondary Subject Heading:	Epidemiology
Keywords:	Childlessness, Infertility, Metabolic syndrome, Diabetes, Register-based cohort study

SCHOLARONE™
Manuscripts

Title: Risk of metabolic disorders in childless men - A population-based cohort study

Authors: Ane Berger Bungum[1,3*], Clara Helene Glazer[1], Jens Peter Bonde[1], Peter M. Nilsson[2], Aleksander Giwercman[3], Sandra Søgaard Tøttenborg[1]

[1] Department of Occupational and Environmental Medicine, Bispebjerg University Hospital, DK-2400 Copenhagen NV, Denmark, [2] Department of Clinical Sciences Malmö, Lund University, Skåne University Hospital, S-20502 Malmö, Sweden, [3] Molecular Reproductive Medicine, Department of Translational Medicine, Lund University, S-21428 Malmö, Sweden

Correspondence and requests for reprints:

*Ane Berger Bungum**

Department of Occupational and Environmental Medicine

Capital Region of Denmark

Bispebjerg - Frederiksberg Hospital

Bispebjerg Bakke 23, Building 20C, 1. sal

DK-2400 Copenhagen NV

Denmark

Telephone: +45 30251797

E-mail: ane.berger.bungum@regionh.dk

For peer review only

Abstract

Objective: To study whether male childlessness is associated with an increased risk of metabolic disorders such as metabolic syndrome (MetS) and diabetes.

Design: A population-based cohort study

Setting: Not applicable.

Participants: 2 572 men from the population-based Malmö Diet and Cancer Cardiovascular Cohort (MDC-CC).

Interventions: None.

Main outcome measure(s): From cross-sectional analyses main outcome measures were odds ratios (OR) and 95% confidence intervals (CI) for MetS and diabetes among childless men. In prospective analyses Hazard ratios (HRs) and 95% CI for diabetes among childless men.

Results: At baseline, in males with a mean age of 57 years, the prevalence of MetS was 26% and 22% among childless men and fathers, respectively. Similarly we observed a higher prevalence of diabetes of 11% among childless men compared to 5% among fathers. In the cross-sectional adjusted analyses childless men had a higher risk of MetS and diabetes, with ORs of 1.22 (95% CI: 0.87;1.72) and 2.12 (95% CI: 1.34;3.36) compared to fathers. In the prospective analysis, during subsequent follow-up, we did not see any additional increase in diabetes risk among childless men.

Conclusion(s): This study provides evidence of an association between male childlessness and a higher risk of MetS and diabetes, which may be due one or more shared risk factors.

For peer review only

Strengths and limitations of this study

- Malmö Diet and Cancer Cardiovascular Cohort (MDC-CC) is sampled from the background urban population, meaning that men from all socio-economic backgrounds were represented.
- This study has the longest mean follow-up among similar studies published.
- Using childlessness as a proxy of infertility may have caused non-differential misclassification
- We were unable to distinguish between fathers of biological or adopted children and fathers of children conceived after in vitro fertilisation (IVF).
- We were not able to distinguish between type 1 and 2 diabetes.

For peer review only

Introduction

A man's reproductive health may not only reflect his chance to become a father, but may also be related to his general health [1]. In recent years, male childlessness and infertility have been reported to be associated with an increased risk of all-cause mortality [2,3], cardiovascular disease [3–5]. Male infertility has also been associated with a higher risk of metabolic disorders [5–7]. Metabolic syndrome (MetS) and type 2 diabetes are metabolic disorders, implying cardiovascular risk, with increasing prevalence worldwide [8,9]. MetS is a syndrome consisting of a cluster of markers, including visceral obesity, hypertension, and hyperglycaemia [8]. Importantly, the syndrome may help to identify individuals at future risk of type 2 diabetes and cardiovascular disease [10–12].

Cross-sectional studies have demonstrated an association between factors related to male reproductive health (e.g. hypogonadism, reduced semen quality and erectile dysfunction) and MetS as well as type 2 diabetes [13–18], but whether poor reproductive health precedes MetS and diabetes or vice versa is uncertain due to the cross-sectional design of these studies. Two recent prospective studies found increased risk of developing diabetes among infertile men [5,7]. Authors of these prospective studies did not suggest a causal relationship for the association, but rather common aetiologies of infertility and diabetes such as shared genetics and factors related to endocrine regulation, lifestyle or *in-utero* exposures. However, these prospective studies failed to adjust for Body Mass Index (BMI), physical activity level, and other lifestyle factors considered as common risk factors for type 2 diabetes and poor male reproductive health [11,19]. Also, as type 2 diabetes can remain asymptomatic and undiagnosed for years, the measure of outcome of both prospective studies have a central limitation in common; dependence on health-seeking behaviour of the study participants.

For peer review only

Assessment of fertility status of a male is based on access to clinical and laboratory data, including semen analysis, which are difficult and costly to obtain in population-based studies. However, information regarding childlessness is readily more easily accessible and may be a feasible proxy for infertility. Therefore, using data from the Malmö Diet and Cancer Cardiovascular Cohort (MDC-CC), we aimed to examine whether male childlessness is associated with MetS and diabetes, while taking potential confounding lifestyle factors into account. We first examined the prevalence of MetS and diabetes in men with and without children, and next we assessed the incidence of diabetes during a follow-up period of – in average - 18 years to study whether the possible association persisted or attenuated later in life. Study participants were investigated for diabetes both at baseline and follow-up clinical examination, limiting the influence of health seeking behaviour and giving reliable estimates of diabetes prevalence and incidence among childless Swedish men.

Materials and methods

Study population

The Malmö Diet and Cancer Cohort (MDC) is a population-based cohort of 28 098 Malmö residents born between 1923 and 1950. During 1991 through 1996, 11 063 men and 17 035 women were enrolled [20]. Following the initial acceptance letter, during the years of 1991-1994, half of the individuals in MDC were invited to participate in a sub-cohort named MDC-Cardiovascular Cohort (MDC-CC). Of these, 2 572 men accepted the invitation (Figure 1).

Both at baseline (1991-1994) and follow-up (2007-2012) MDC-CC participants completed a questionnaire regarding marital status, number of children, and lifestyle factors. They also underwent a clinical examination including body composition, blood pressure measurement and

For peer review only

collection of venous blood samples [20]. At follow-up examination where 1 522 men participated, the clinical examination also included an oral glucose tolerance test (OGTT) in study participants without known diabetes.

Ethical Approval

The MDC project was approved by the Ethics Committee of the Lund University (LU 51-90) and by the Swedish Data Inspection Agency.

Information on childlessness

Information regarding childlessness came from two sources: the baseline questionnaire and the Swedish Tax Agency (STA). In the questionnaire, participants were asked *"Do you have any children?"* with reply options 'Yes' or 'No'. The STA holds the number of registered children and their respective birth dates. Data were linked using the unique 10-digit personal identification number assigned to all Swedish citizens. We linked these data sources to stratify the participants into four groups; 'Childless', 'One or more child', 'Conflicting information', and 'Unknown'. 'Conflicting information' appeared if a participant answered "No" to *"Do you have any children?"* in the baseline questionnaire, but was registered with one or more children at the STA. Men with 'Conflicting information' who became fathers after the entry date into the MDC-CC cohort were treated as 'Childless' as they were childless at baseline. The remaining men with 'Conflicting information' were registered as fathers in the registry of STA, and were therefore treated as having 'One or more child'. We used the designation "Unknown" if no information regarding children was available from the STA and if no answer was provided to *"Do you have any children?"* in the baseline questionnaire.

For peer review only

Assessment of MetS

MetS was defined according to the harmonized criteria of the International Diabetes Federation Task Force on Epidemiology and Prevention; National Heart, Lung, and Blood Institute; American Heart Association; World Heart Federation; International Atherosclerosis Society; and International Association for the Study of Obesity [21]. Accordingly, MetS was present if three or more of the follow criteria were met: Fasting blood glucose (fB-glucose) level above 5.6 mmol/L or the use of anti-diabetic drugs, High Density Lipoprotein (HDL) cholesterol level below 1.03 mmol/L or the use of lipid modifying treatment, triglycerides level above 1.7 mmol/L or the use of lipid-lowering drugs, waist circumference higher than 102 cm and blood pressure above 130/85 mmHg, or the use of antihypertensive medicine [21]. Data regarding MetS criteria were only available from the baseline clinical examination.

Diagnosis of diabetes

The diabetes cases and the date of diagnosis were identified from 14 different data sources, including the Swedish National Diabetes register (NDR), The Swedish Prescribed Drug Register, and from baseline and follow-up screenings in MDC, MDC-CC, and the Malmö Preventive Project (MPP) [22]. These sources were used to identify prevalent cases of diabetes (type 1 and type 2) at baseline, and new-onset incident cases of diabetes (type 1 and type 2) during the follow-up period. In brief, individuals with a date of diagnosis registered in the NDR and/or Diabetes 2000 Register were considered to have diabetes. The same was true for individuals in the local HbA_{1c} Register in Malmö with at least two $HbA_{1c} \geq 6\%$, those with the Tenth Edition of International Classification of Diseases (ICD10) codes E10-E14 and O244-O249, and corresponding ICD7-9 codes in the National Hospitalisation Register or in the Cause-of-death Register, and men in The Swedish Prescribed Drug Register with Anatomical Therapeutic Chemical (ATC) code A10.

For peer review only

From baseline questionnaires of MDC and MPP, participants who answered "Yes" to *"Do you have diabetes?"* and/or listed antidiabetic drugs were considered as patients with diabetes. At the baseline examination of MDC-CC, individuals were considered having diabetes if the fasting blood glucose (fB-glucose) measurement was ≥ 6.5 mmol/L. In MPP and at the MDC-CC follow-up, fB-glucose ≥ 6.5 mmol/L had to be verified through OGTT and or fP-glucose measurements. A full list of the diabetes diagnostic sources is provided in supplementary Table I. The participants were considered as having diabetes from their first diagnosis of diabetes while any and subsequent contradictory information about diabetes was ignored [23].

Statistical analyses

Cross-sectional analysis

To assess the association between male childlessness and metabolic disorders, the prevalence of MetS and diabetes at baseline 1991-1996 was compared among men with and without children by means of logistic regression and reported as odds ratios (OR) with corresponding 95% confidence intervals (CI). As childlessness could be a function of not having a partner, sensitivity analyses including only married men were also performed.

Prospective analysis

To assess whether the possible association between male childlessness and diabetes persisted or attenuated later in life, hazard ratios (HR) with 95% CIs of diabetes among childless men compared to fathers were computed using Cox proportional hazard models. Men with pre-existing diabetes at baseline were excluded. The men were followed from enrolment into the MDC-CC until date of diabetes diagnosis, emigration, death, or end of follow-up 31st of December 2014. Kaplan-Meier

For peer review only

plots allowed for visual evaluation of proportional hazards assumption. As with the cross-sectional analysis, we completed a sensitivity analysis including only married men.

Potential confounders were identified *a priori* using directed acyclic graphs [24]. Adjustments were performed in two steps in all analyses. The first adjustment step (*Model I*) included age in years, marital status (Married/Unmarried/Divorced/Widower), Socio-economic index (SEI) (Workers, unskilled/Workers, skilled/Lower positioned official or salaried/Intermediate positioned official or salaried/Employers or self-employed), and highest level of education attained (No education/Primary school/Secondary school/High school/>One year education after high school/University degree). The second adjustment step (*Model II*) also included BMI in kg/m^2, alcohol consumption in grams per day, smoking habits (Regularly smoker/Occasional smoker/Stopped smoking/Never smoked) and physical activity level in minutes per week. The reason for the distinction between *Model I* and *Model II* is that BMI, alcohol consumption, smoking habits, and physical activity level may confound as well as mediate the association between childlessness, MetS and diabetes. For instance, smoking habits can affect a man's reproductive health, but on the contrary if a man does become a father, this can affect his smoking habits. In the statistical analyses, probability values (*p*-values) were not included, instead 95% CI for measures of association were reported to display the measure of precision [25]. All statistical tests were performed using SAS version 9.4.

Results

Among all 2 572 men, 422 had missing information regarding fatherhood status and were excluded from analyses. Twenty men had 'Conflicting information' regarding fatherhood, of which 18 men had registered children in the STA before baseline, and 2 men became fathers after baseline and

For peer review only

thus treated as 'Childless'. Consequently, 2 150 men were included in the analyses of which 15% were childless and 85% fathers (Table I). The mean age in the cohort was 57 years at baseline. The baseline socio-demographic and lifestyle characteristics were equally distributed in general among childless men and fathers, except for the distribution of marital status, with 81% of fathers being married compared to only 37% of childless men being married.

Association between childlessness, MetS, and diabetes

<u>MetS</u>

At baseline, with the mean age of 57 years, we identified 26% MetS cases among childless men and 22% MetS cases among fathers. The major contributing factor for MetS among childless men was hyperglycaemia (Table II). The fully adjusted analyses (*Model II*) indicated a non-significant trend for an increased risk of MetS in childless men compared to fathers, as OR was 1.22 (95% CI: 0.87;1.72) (Table III). When comparing married childless men to married fathers the association became stronger and significant with OR 1.62 (95% CI: 1.01; 2.60) in the fully adjusted model (Table III).

<u>Diabetes</u>

The prevalence of diabetes at baseline, was 11% among childless men and 5% among fathers. In the fully adjusted analysis (*Model II*) childless men had a higher risk of diabetes with OR 2.12 (95% CI: 1.34;3.36) (Table IV). The association persisted when comparing married fathers to married childless men (Table IV).

Risk of developing diabetes

For peer review only

The incidence of diabetes was 20% among childless men and 22% among fathers. The mean follow up time was 18.3 years. In the fully adjusted analysis (Model II) the result showed no trend of an association between childlessness and incident diabetes (Table V). However, results from analyses including only married men showed a non-significant trend for an association with HR 1.13 (95% CI: 0.74;1.73) (Table V).

Discussion

Main findings

Our study demonstrates cross-sectional associations between male childlessness, MetS and diabetes. As expected, associations were generally stronger in analyses restricted to married men where a lack of reproductive opportunities can have been accounted for. Our findings could not be attributed to differences in lifestyle or socio-demographic characteristics between childless men and fathers.

Prior literature

Our findings are comparable to other studies finding higher rates of medical co-morbidities, poorer general health status, and type 2 diabetes among infertile men [15,26–28]. MetS and diabetes, with hyperglycemia as a central symptom in common, have been suggested to affect the endocrine control of male reproductive function, and to impair spermatogenesis, sperm maturation, erectile function and ejaculation [11,13,29], and the major contributing factor for MetS among our cohort of childless men was hyperglycaemia. However, conflicting results regarding the impact of MetS and diabetes on semen quality parameters exists [13,30]. Also, the incidence of diabetes and MetS is low among the general infertile population, due to the low incidence of diabetes and MetS among

For peer review only

men in their reproductive years [6,13,31], and therefore, we do not assume this causal relationship between infertility, MetS and diabetes to explain the entire association found in the present study. Our cross-sectional findings are also consistent with reports from the U.S. [5] and Denmark [7] in which male factor infertility was associated with a 30-45% higher risk of diabetes. Findings of these prospective studies are also supported by a recent Danish study [32] which found higher hospitalization rates for diabetes among men with poor semen quality and the authors of these latter mentioned studies suggested common aetiologies for male infertility, poor semen quality and diabetes. However, our prospective results which showed no additional increase in risk can seem contradictory, but the mean age of the study population in the latter mentioned studies was more than 20 years lower than in the present study.

Mechanisms

The relationship between male reproductive and somatic health is rather complex and different causal mechanisms and common aetiologies have been proposed for the association, namely shared genetic origins, *in utero*-, hormonal- or environmental/lifestyle factors [1,2,5,7,32]. More than 150 genes are linked to both male infertility and simultaneously involved in pathways important for several diseases, such as cancer, cardiovascular, and metabolic disorders [33]. Also, as male germ cell differentiation includes expression of up to 4% of all mammalian genes, it seems plausible that mutations in these genes could cause or contribute both to infertility, MetS as well as diabetes [34]. Further, as our cross-sectional results display a strong increase in risk of diabetes until the mean age of 57 years, and no additional increase in diabetes risk hereafter, as seen in our prospective results, this points to early onset of diabetes and support the hypothesis of genetic origins, as genetic predisposition increases the risk of early onset type 2 diabetes [35]. In addition to genetic defects, maternal health behaviour and environmental exposures during pregnancy have been hypothesized

For peer review only

to act directly or through epigenetic mechanisms on the foetus and thereby influence both male reproductive health and somatic health [2,36].

Low testosterone levels have also been associated with infertility and an increased risk of MetS and diabetes, as testosterone plays an import role in glucose and lipid metabolism [37–39]. One study found low testosterone to predict development of MetS and onset of diabetes, even after adjusting for BMI, insulin resistance and other established risk factors for these conditions [38,40]. Another study among men with infertility problems and decreased sperm counts, reported low testosterone levels to be associated with higher levels of HbA_{1c} [41]. However, obesity, which is a strong risk factor for MetS and diabetes, is also known to lower testosterone levels, as testosterone is converted to oestradiol in adipose tissue. On the other hand, low testosterone levels are also known to increase obesity [42]. The causality of the relationship between low testosterone, MetS and diabetes, is therefore unclear but may be bidirectional [37].

Lastly, low socio-economic status and adverse lifestyle factors have been suspected to explain the association between poor reproductive and somatic health. In the present study we did adjust for multiple lifestyle factors and socio-economic status, and associations were still seen. This provides further support to the hypotheses of shared genetic origins, *in utero*-, or hormonal factors as common aetiologies for male infertility and metabolic disorders. Nevertheless, the association of male childlessness, MetS and diabetes could be results of other environmental, hormonal, social or lifestyle factors related to childlessness that we did not adjust for.

Strengths and limitations

The present study has several strengths. *Firstly,* the MDC-CC is sampled from the background urban population, meaning that men from all socio-economic backgrounds were represented. A previous health survey study from the city of Malmö with a 75% participation rate in corresponding

For peer review only

age groups showed no difference in baseline socio-demographic characteristics compared to study participants in the MDC [20]. Also, among Swedish men born between 1935 and 1945 one in six remained childless [43], which is comparable with the proportion of childless men in our cohort. *Secondly*, the mean cohort age at baseline was already advanced, and childlessness at an advanced age strengthens the assumption of infertility. *Thirdly*, the valid and comprehensive national Swedish registers provide information on emigrations, death, and disease limited loss to follow-up and made long-term follow up of more than 18 years in average possible – the longest mean follow-up among the few similar studies published to date [5,7,32].

Our study also has limitations. Data regarding MetS criteria were only available from the baseline clinical examination, why a prospective analysis of the association between childlessness and MetS could not be performed. We were not able to distinguish between type 1 and 2 diabetes. However, as type 2 diabetes accounts for 85-90% of all diabetes cases in Sweden [44] and the men were middle-aged at inclusion we expect most cases to have been type 2 diabetes. Furthermore, using childlessness as a proxy of infertility may pose some challenges. The group of childless men is heterogeneous in relation to different causes of childlessness (e.g. voluntarily childlessness, homosexual men, men with infertile partners, or single men). As only one in four childless Swedish men reported childlessness to be volitional [43], this limits the risk of our sample to reflect voluntary childlessness. However, more than 50% of the childless men in our cohort reported to be unmarried, which can have limited the reproductive opportunities for this group. To account for such differences in reproductive opportunities, sensitivity analyses including only married men were performed and associations then became generally stronger. Yet, 40-50% of infertility cases among couples are due to male factor infertility [45], which means that up to 50% of the men in our group of childless could be fathers if they had a fertile partner. We do not expect childlessness to be

For peer review only

influenced by the disease. Thus we expect that exposure misclassification will be non-differential, but we don't assume this to have influenced our estimate considerably.

Another drawback was the inability to distinguish between fathers of biological or adopted children and fathers of children conceived after in vitro fertilisation (IVF). However, as adoption rates in Sweden were rather low at the time of baseline [46], misclassification would attenuate the estimate towards the null. Likewise, the chance of having children conceived after IVF was insignificant as assisted reproduction as treatment of impaired male fertility was not widely performed before and at the time of baseline in 1991-1994 [47]. This strengthens the usefulness of childlessness as a proxy for infertility in the present cohort. Nevertheless, the lacking ascertainment of male infertility among childless men and the resulting non-differential misclassification is problematic, but we did find an association between male childlessness and metabolic disorders, which previously have been found among infertile men.

Conclusion

In conclusion, our study showed a higher risk of MetS and diabetes among childless middle-aged men that could not be explained by differences in lifestyle, socio-demographic characteristics, or health seeking behaviour. This supports the hypothesis that a man's reproductive health is closely intertwined with his somatic health, and of underlying common aetiologies such as prenatal, genetic, and hormonal factors. While using childlessness as a proxy of male infertility may cause some bias due to non-differential misclassification, it may still provide insight into a man's risk of disease. The simple objective measure of exposure enables for future studies to examine the association between male reproductive health and somatic health in large population-based cohorts.

For peer review only

Funding

This study was funded by ReproUnion and also supported by the Medical Research Council of Sweden (grant K2011-65X-20752-04-6), the Region Skåne County Council, the Ernhold Lundstrom Foundation, for the MDC-CC follow-up clinical examination.

Conflict of interest

None declared.

Author's contribution

J.P.B. and A.G. acquired funding for the study. A.B.B., C.H.G., S.S.T., A.G. and J.P.B. designed the study. A.B.B, S.S.T and C.H.G. analyzed data and A.B.B. wrote the manuscript. P.N. contributed with acquisition of data, critical discussion and revision of the paper. All authors contributed to data analysis/interpretation, critical revision of the paper and final approval of the manuscript.

Data sharing statement

No additional unpublished data.

For peer review only

References

1 Glazer CH, Bonde JP, Eisenberg ML, *et al.* Male Infertility and Risk of Nonmalignant Chronic Diseases: A Systematic Review of the Epidemiological Evidence. *Semin Reprod Med* 2017;**35**:282–90. doi:10.1055/s-0037-1603568

2 Jensen TK, Jacobsen R, Christensen K, *et al.* Good Semen Quality and Life Expectancy: A Cohort Study of 43,277 Men. *Am J Epidemiol* 2009;**170**:559–65. doi:10.1093/aje/kwp168

3 Ringbäck Weitoft G, Burström B, Rosén M. Premature mortality among lone fathers and childless men. *Soc Sci Med* 2004;**59**:1449–59. doi:10.1016/j.socscimed.2004.01.026

4 Eisenberg ML, Park Y, Hollenbeck AR, *et al.* Fatherhood and the risk of cardiovascular mortality in the NIH-AARP Diet and Health Study. *Hum Reprod* 2011;**26**:3479–85. doi:10.1093/humrep/der305

5 Eisenberg ML, Li S, Cullen MR, *et al.* Increased risk of incident chronic medical conditions in infertile men: analysis of United States claims data. *Fertil Steril* 2016;**105**:629–36. doi:10.1016/j.fertnstert.2015.11.011

6 Ventimiglia E, Capogrosso P, Serino A, *et al.* Metabolic syndrome in White-European men presenting for secondary couple's infertility: an investigation of the clinical and reproductive burden. *Asian J Androl* 2016;**19**:368–73. doi:10.4103/1008-682X.175783

7 Glazer CH, Bonde JP, Giwercman A, *et al.* Risk of diabetes according to male factor infertility: A register-based cohort study. *Hum Reprod* 2017;:[Epub ahead of print]. doi:10.1093/humrep/dex097

8 Alberti KGM, Zimmet P, Shaw J. The metabolic syndrome—a new worldwide definition. Lancet. 2005;**366**:1059–62. doi:10.1016/S0140-6736(05)67402-8

9 World Health Organization (WHO). Global report on diabetes. 2016. http://www.who.int/diabetes/global-report/en/ (accessed 18 Feb 2017).

10 Vliet-Ostaptchouk JV van, Nuotio M-L, Slagter SN, *et al.* The prevalence of metabolic syndrome and metabolically healthy obesity in Europe: a collaborative analysis of ten large cohort studies. *BMC Endocr Disord* 2014;**14**. doi:10.1186/1472-6823-14-9.

11 Kasturi SS, Tannir J, Brannigan RE. The Metabolic Syndrome and Male Infertility. *J Androl* 2008;**29**:251–9. doi:10.2164/jandrol.107.003731

12 Okoloise M. The metabolic syndrome as predictor of type 2 diabetes: The San Antonio Heart Study. *Diabetes Care* 2003;**26**:3153–9.

13 Ventimiglia E, Capogrosso P, Colicchia M, *et al.* Metabolic syndrome in white European

For peer review only

men presenting for primary couple's infertility: investigation of the clinical and reproductive burden. *Andrology* 2016;**4**:944–51. doi:10.1111/andr.12232

14 Delfino M, Imbrogno N, Elia J, *et al.* Prevalence of diabetes mellitus in male partners of infertile couples. *Minerva Urol Nefrol* 2007;**59**:131–5.

15 Bener A, Al-Ansari AA, Zirie M, *et al.* Is male fertility associated with type 2 diabetes mellitus? *Int Urol Nephrol* 2009;**41**:777–84. doi:10.1007/s11255-009-9565-6

16 Giugliano F, Maiorino M, Bellastella G, *et al.* Determinants of erectile dysfunction in type 2 diabetes. *Int J Impot Res* 2010;**22**:204–9. doi:10.1038/ijir.2010.1

17 Dunsmuir WD, Holmes SAV. The Aetiology and Management of Erectile, Ejaculatory, and Fertility Problems in Men with Diabetes Mellitus. *Diabet Med* 1996;**13**:700–8. doi:10.1002/(SICI)1096-9136(199608)13:8<700::AID-DIA174>3.0.CO;2-8

18 Laaksonen DE, Niskanen L, Punnonen K, *et al.* Sex hormones, inflammation and the metabolic syndrome: a population-based study. *Eur J Endocrinol* 2003;**149**:601–8. doi:10.1530/EJE.0.1490601

19 Jensen T, Anderson A, Jorgensen N, *et al.* Body mass index in relation to semen quality and reproductive hormonesamong 1,558 Danish men. *Fertil Steril* 2004;**82**:863–70. doi:10.1016/j.fertnstert.2004.03.056

20 Manjer J, Carlsson S, Elmståhl S, *et al.* The Malmö diet and cancer study: representativity, cancer incidence and mortality in participants and non-participants. *Eur J Cancer Prev* 2001;**10**:489–99.

21 Alberti KGM, Eckel RH, Grundy SM, *et al.* Harmonizing the Metabolic Syndrome. *Circulation* 2009;**120**:1640–5.

22 Magnusson M, Wang TJ, Clish C, *et al.* Dimethylglycine Deficiency and the Development of Diabetes. *Diabetes* 2015;**64**:3010–6. doi:10.2337/db14-1863

23 Borné Y, Smith JG, Nilsson PM, *et al.* Total and Differential Leukocyte Counts in Relation to Incidence of Diabetes Mellitus: A Prospective Population-Based Cohort Study. *PLoS One* 2016;**11**:e0148963. doi:10.1371/journal.pone.0148963

24 Greenland S, Pearl J, Robins JM. Causal Diagrams for Epidemiologic Research. *Epidemiology* 1999;**10**:37–48.

25 Rigby AS. Getting past the statistical referee: moving away from P-values and towards interval estimation. *Health Educ Res* 1999;**14**:713–5. doi:10.1093/her/14.6.713

26 Ventimiglia E, Capogrosso P, Boeri L, *et al.* Infertility as a proxy of general male health:

For peer review only

results of a cross-sectional survey. *Fertil Steril* 2015;**104**:48–55. doi:10.1016/j.fertnstert.2015.04.020

27 Salonia A, Matloob R, Gallina A, *et al.* Are Infertile Men Less Healthy than Fertile Men? Results of a Prospective Case-Control Survey. *Eur Urol* 2009;**56**:1025–32. doi:10.1016/j.eururo.2009.03.001

28 Eisenberg ML, Li S, Behr B, *et al.* Relationship between semen production and medical comorbidity. *Fertil Steril* 2015;**103**:66–71. doi:10.1016/j.fertnstert.2014.10.017

29 Demir T, Demir O, Kefi A, *et al.* Prevalence of erectile dysfunction in patients with metabolic syndrome. *Int J Urol* 2006;**13**:385–8. doi:10.1111/j.1442-2042.2006.01310.x

30 Vignera S La, Condorelli R, Vicari E, *et al.* Diabetes Mellitus and Sperm Parameters. *J Androl* 2012;**33**:145–53. doi:10.2164/jandrol.111.013193

31 Sexton WJ, Jarow JP. Effect of diabetes mellitus upon male reproductive function. *Urology* 1997;**49**:508–13. doi:10.1016/S0090-4295(96)00573-0

32 Latif T, Kold Jensen T, Mehlsen J, *et al.* Semen quality is a predictor of subsequent morbidity. A Danish cohort study of 4,712 men with long-term follow-up. *Am J Epidemiol* 2017;:[Epub ahead of print]. doi:10.1093/aje/kwx067

33 Tarín JJ, García-Pérez MA, Hamatani T, *et al.* Infertility etiologies are genetically and clinically linked with other diseases in single meta-diseases. *Reprod Biol Endocrinol* 2015;**13**. doi:10.1186/s12958-015-0029-9

34 Matzuk MM, Lamb DJ. The biology of infertility: research advances and clinical challenges. *Nat Med* 2008;**14**:1197–213. doi:10.1038/nm.f.1895

35 Wilmot E, Idris I. Early onset type 2 diabetes: risk factors, clinical impact and management. *Ther Adv Chronic Dis* 2014;**5**:234–44. doi:10.1177/2040622314548679

36 Skakkebaek NE, Rajpert-De Meyts E, Buck Louis GM, *et al.* Male Reproductive Disorders and Fertility Trends: Influences of Environment and Genetic Susceptibility. *Physiol Rev* 2016;**96**:55–97.

37 Kelly DM, Jones TH. Testosterone: A metabolic hormone in health and disease. *J Endocrinol* 2013;**217**:R25-45. doi:10.1530/JOE-12-0455

38 Laaksonen DE, Niskanen L, Punnonen K, *et al.* Testosterone and sex hormone-binding globulin predict the metabolic syndrome and diabetes in middle-aged men. *Diabetes Care* 2004;**27**:1036–41. doi:10.2337/diacare.27.5.1036

39 Brand JS, Rovers MM, Yeap BB, *et al.* Testosterone, sex hormone-binding globulin and the

For peer review only

metabolic syndrome in men: An individual participant data meta-analysis of observational studies. *PLoS One* 2014;**9**:e100409. doi:10.1371/journal.pone.0100409

40 Li C, Ford ES, Li B, *et al.* Association of Testosterone and Sex Hormone-Binding Globulin With Metabolic Syndrome and Insulin Resistance in Men. *Diabetes Care* 2010;**33**:1618–24. doi:10.2337/dc09-1788

41 Bobjer J, Bogefors K, Isaksson S, *et al.* High prevalence of hypogonadism and associated impaired metabolic and bone mineral status in subfertile men. *Clin Endocrinol (Oxf)* 2016;**85**:189–95. doi:10.1111/cen.13038

42 Kelly DM, Jones TH. Testosterone and obesity. *Obes Rev* 2015;**16**:581–606. doi:10.1111/obr.12282

43 Statistics Sweden (SCB). Childbearing patterns of different generations. 2011. http://www.scb.se/statistik/_publikationer/BE0701_2011A01_BR_BE51BR1103.pdf (accessed 28 Jun 2017).

44 Eliasson M, Boström G. Chapther 5.2: Major public health problems – diabetes. *Scand J Public Heal Suppl* 2006;**67**:59–68. doi:10.1080/14034950600676883

45 Kumar N, Singh AK. Trends of male factor infertility, an important cause of infertility: A review of literature. *J Hum Reprod Sci* 2015;**8**:191–6. doi:10.4103/0974-1208.170370

46 Statistics Sweden (SCB). Internationella adoptioner minskar. 2013.http://www.scb.se/sv_/Hitta-statistik/Artiklar/Internationella-adoptioner-minskar/ (accessed 6 Jun 2017).

47 Gottvall K, Lundqvist E, Källén K. Graviditeter, förlossningar och nyfödda barn. Medicinska födelseregistret 1973-2014. Assisterad befruktning 1991-2013. 2015.http://www.socialstyrelsen.se/publikationer2015/2015-12-27 (accessed 15 May 2017).

For peer review only

Table I: Socio-demographic and lifestyle characteristics of men with and without children at baseline. Means (SD) and proportions

	**Childless men (*n*=333)**	**Fathers (*n*=1 817)**
**Age (years)** (*n*=2 150)	57.3 (6.1)	57.1 (5.9)
**Marital status** (*n*=2 052)		
- **Married (%)**	37	81
- **Unmarried (%)**	51	3
- **Divorced (%)**	8	14
- **Widower (%)**	4	2
**Socio-economic index** (*n*=1 927)		
- **Employers and self-employed (%)**	16	22
- **Official/Salaried, intermediate position (%)**	21	24
- **Official/Salaried, lower position (%)**	21	15
- **Workers, skilled (%)**	17	21
- **Workers, unskilled (%)**	25	18

For peer review only

For peer review only - http://bmjopen.bmj.com/site/about/guidelines.xhtml

**Highest level of education** (*n*=2 056)		
- **No education (%)**	0	1
- **Primary school (%)**	50	45
- **Secondary school (%)**	22	20
- **High school (%)**	11	12
- **>1 year education after high school (%)**	7	10
- **University degree (%)**	10	12
**BMI (kg/m^2)** (*n*=2 148)	26 (4.1)	26.1 (3.3)
**Alcohol (g/day)** (*n*=2 057)	14.6 (18.4)	15.8 (15.2)
**Present smoker** (*n*=2 058)		
- **Regularly (%)**	24	22
- **Occasionally (%)**	4	5
- **Stopped (%)**	38	43
- **Never (%)**	34	30
**Physical activity score (minutes/week)** (*n*=2 041)	8 675.3 (6 771.0)	8 361.9 (6 224.9)
*SD (standard deviation)		

For peer review only

Table II: Odds ratio (OR) with 95% confidence intervals (95% CI) of metabolic syndrome (MetS) components in childless men compared to fathers at baseline

	**Childless men (n=333)**		**Fathers (n=1 817)**	
	***n* cases**	**OR [95% CI]**	***n* cases**	**OR [95% CI]**
**Hyperglycaemia ***	88	1.59 [1.21;2.08]	335	1 (ref)
**Hypo-HDL cholesterolemia †**	102	0.78 [0.60;1.01]	670	1 (ref)
**Hyperlipidemia ‡**	97	1.04 [0.80;1.34]	515	1 (ref)
**Waist circumference >102 cm**	54	1.11 [0.81;1.53]	270	1 (ref)
**Hypertension §**	222	1.13 [0.88;1.45]	1160	1 (ref)

* Hyperglycemia defined as a fasting blood glucose level ≥ 5.6 mmol/L or by the use of anti-diabetic medicine

† Hypo-HDL cholesterolemia defined as HDL<1.03 mmol/L or by the use of drug treatment

‡ Hyperlipidemia defined as Triglycerides≥1.7 mmol/L or by the use of lipid lowering drugs

§ Hypertension (elevated blood pressure) defined by ≥130/85 mmHg or by the use of antihypertensive drugs

For peer review only - http://bmjopen.bmj.com/site/about/guidelines.xhtml

Table III: Odds ratio (OR) with 95% confidence intervals (95% CI) for MetS in childless men relative to fathers

**Total population (*n*=2 150)**				
		**Crude**	**Model I ***	**Model II †**
	***n* cases**	**OR [95% CI]**	**OR [95% CI]**	**OR [95% CI]**
**Fathers (*n*=1 817)**	402 (22.1%)	1 (ref)	1 (ref)	1 (ref)
**Childless men (*n*=333)**	85 (25.5%)	1.21 [0.92;1.58]	1.22 [0.92;1.64]	1.22 [0.87;1.72]
**Only married men (*n*=1 515)**				
		**Crude**	**Model I ‡**	**Model II §**
	***n* cases**	**OR [95% CI]**	**OR [95% CI]**	**OR [95% CI]**
**Fathers (*n*=1 392)**	317 (22.8%)	1 (ref)	1 (ref)	1 (ref)
**Childless men (*n*=123)**	42 (34.2%)	1.76 [1.19;2.61]	1.66 [1.11;2.49]	1.62 [1.01;2.60]

* Model 1: Adjusted for age, marital status, SEI and education (*n*=1 923)

† Model 2: Adjusted for age, marital status, SEI, education, BMI, alcohol (g/day), smoking and physical activity score (*n*=1 895)

‡ Model I: Adjusted for age, SEI and education (*n*=1 414)

§ Model II: Adjusted for age, SEI, education, BMI, alcohol (g/day), smoking and physical activity score (*n*=1 396)

For peer review only - http://bmjopen.bmj.com/site/about/guidelines.xhtml

Table IV: Odds ratio (OR) with 95% confidence intervals (95% CI) for diabetes in childless men relative to men with children

**Total population (*n*=2 150)**				
		**Crude**	**Model I ***	**Model II †**
	***n* cases**	**OR [95% CI]**	**OR [95% CI]**	**OR [95% CI]**
**Fathers (*n*=1 817)**	87 (4.8%)	1 (ref)	1 (ref)	1 (ref)
**Childless men (*n*=333)**	35 (10.5%)	2.34 [1.55;3.52]	2.26 [1.44;3.54]	2.12 [1.34;3.36]
**Only married men (*n*=1 515)**				
		**Crude**	**Model I ‡**	**Model II §**
	***n* cases**	**OR [95% CI]**	**OR [95% CI]**	**OR [95% CI]**
**Fathers (*n*=1 392)**	64 (4.6%)	1 (ref)	1 (ref)	1 (ref)
**Childless men (*n*=123)**	13 (10.6%)	2.45 [1.30;4.59]	2.29 [1.18;4.43]	2.05 [1.03;4.08]

* Model I: Adjusted for age, marital status, SEI and education (*n*=1 923)

† Model II: Adjusted for age, marital status, SEI, education, BMI, alcohol (g/day), smoking and physical activity score (*n*=1 895)

‡ Model I: Adjusted for age, SEI and education (*n*=1 414)

§ Model II: Adjusted for age, SEI, education, BMI, alcohol (g/day), smoking and physical activity score (*n*=1 396)

For peer review only

Table V: Hazard ratio (HR) with 95% confidence intervals (95% CI) for diabetes in childless men relative to fathers

**Total population (*n*=2 028)**				
		**Crude**	**Model I ***	**Model II †**
	***n* cases**	**HR [95% CI]**	**HR [95% CI]**	**HR [95% CI]**
**Fathers (n=1 730)**	373 (21.6%)	1 (ref)	1 (ref)	1 (ref)
**Childless men (n=298)**	60 (20.1%)	1.05 [0.80;1.38]	1.12 [0,85;1.49]	1.02 [0.76;1.37]
**Only married men (*n*=1 438)**				
		**Crude**	**Model I ‡**	**Model II §**
	***n* cases**	**HR [95% CI]**	**HR [95% CI]**	**HR [95% CI]**
**Fathers (n=1 328)**	281 (21.2%)	1 (ref)	1 (ref)	1 (ref)
**Childless men (n=110)**	24 (21.8%)	1.17 [0.77;1.77]	1.20 [0,79;1.83]	1.13 [0.74;1.73]

* Model I: Adjusted for age, marital status, SEI, and education (*n*=1 820)

† Model II: Adjusted for age, marital status, SEI, education, BMI, alcohol (g/day), smoking, and physical activity score (*n*=1 794)

‡ Model I: Adjusted for age, SEI and education (*n*=1 346)

§ Model II: Adjusted for age, SEI, education, BMI, alcohol (g/day), smoking and physical activity score (*n*=1 330)

For peer review only

Figure 1: Malmö diet cancer cardiovascular cohort (MDC-CC) recruitment

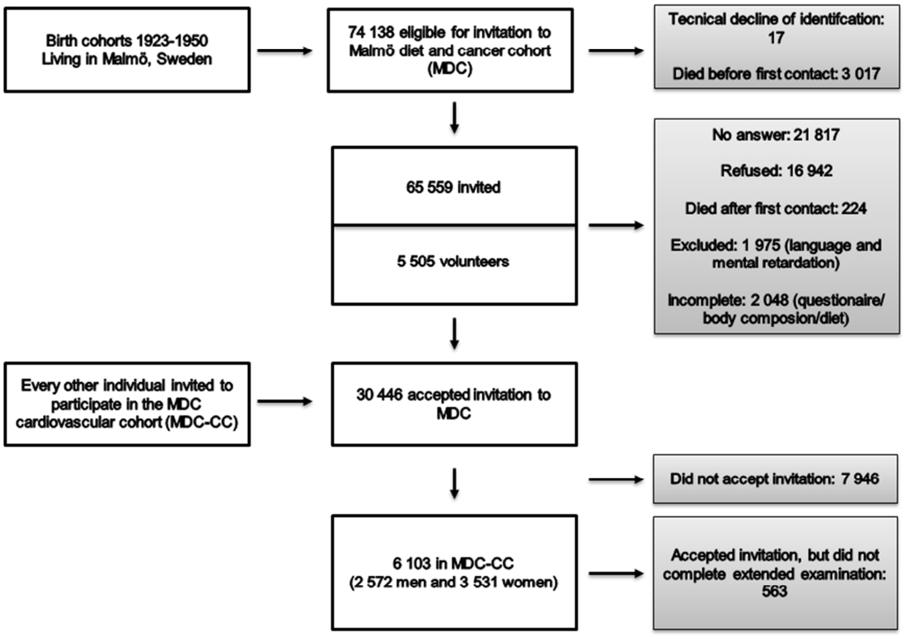

Figure 1

203x152mm (120 x 120 DPI)

Supplementary table I: Fourteen different sources of data were used to identify diabetic cases among men included in MDC-CC.

**Source of data**	**Diabetes criteria**
The Swedish National Diabetes Register (NDR)	All registered individuals
The Diabetes 2000 Registry	All registered individuals
The HbA1c register at Clinical Chemistry, Malmö	Individuals with at least two HbA1c ≥ 6% (not on the same day)
The Swedish Hospital Discharge Register (also The National Inpatient Register (IPR))	Individuals with the ICD10 codes E10-E14 and O244-O249 (corresponding ICD7-9 codes)
The Swedish National Patient Register – Outpatient Care	Individuals with the ICD10 codes E10-E14 and O244-O249 (corresponding ICD7-9 codes)
The Swedish Cause-of-death Register	Individuals with the ICD10 codes E10-E14 and O244-O249 (corresponding ICD7-9 codes)
The Swedish Prescribed Drug Register	Individuals with ATC code A10
MPP baseline screening (1974-92)	Based on questionnaire, fB-glucose ≥ 6.5 mmol/L and glucose ≥ 11 mmol/L at 120 minutes OGTT
MPP 6-year rescreening (1981-89)	Based on questionnaire, fB-glucose ≥ 6.5 mmol/L and glucose ≥ 11 mmol/L at 120 minutes OGTT
MPP rescreening (2002-06)	Based on questionnaire, list of antidiabetic drugs and fP-glucose ≥ 7 mmol/L
MDC baseline screening (1991-96)	Based on questionnaire and list of antidiabetic drugs
MDC cardiovascular cohort baseline screening (1992-94)	Based on fB-glucose ≥ 6.5 mmol/L
MDC 5-year rescreening (1997-2001)	Based on questionnaire and list of antidiabetic drugs

For peer review only

MDC cardiovascular cohort rescreening (2007-12)	Based on questionnaire, list of antidiabetic drugs, fB-glucose ≥ 6.5 mmol/L verified by fP-glucose ≥ 7 mmol/L and glucose ≥ 11 mmol/L at 120 minutes OGTT

For peer review only

STROBE Statement—checklist of items that should be included in reports of observational studies

	Item No	Recommendation	
**Title and abstract**	1	(*a*) Indicate the study's design with a commonly used term in the title or the abstract	✓ page 1-3
		(*b*) Provide in the abstract an informative and balanced summary of what was done and what was found	✓
**Introduction**			page 4-5
Background/rationale	2	Explain the scientific background and rationale for the investigation being reported	✓
Objectives	3	State specific objectives, including any prespecified hypotheses	✓
**Methods**			page 5-9
Study design	4	Present key elements of study design early in the paper	✓
Setting	5	Describe the setting, locations, and relevant dates, including periods of recruitment, exposure, follow-up, and data collection	✓
Participants	6	(*a*) *Cohort study*—Give the eligibility criteria, and the sources and methods of selection of participants. Describe methods of follow-up	✓
		Case-control study—Give the eligibility criteria, and the sources and methods of case ascertainment and control selection. Give the rationale for the choice of cases and controls	
		Cross-sectional study—Give the eligibility criteria, and the sources and methods of selection of participants	✓
		(*b*) *Cohort study*—For matched studies, give matching criteria and number of exposed and unexposed	✓
		Case-control study—For matched studies, give matching criteria and the number of controls per case	
Variables	7	Clearly define all outcomes, exposures, predictors, potential confounders, and effect modifiers. Give diagnostic criteria, if applicable	✓
Data sources/ measurement	8*	For each variable of interest, give sources of data and details of methods of assessment (measurement). Describe comparability of assessment methods if there is more than one group	✓
Bias	9	Describe any efforts to address potential sources of bias	✓
Study size	10	Explain how the study size was arrived at	✓
Quantitative variables	11	Explain how quantitative variables were handled in the analyses. If applicable, describe which groupings were chosen and why	✓
Statistical methods	12	(*a*) Describe all statistical methods, including those used to control for confounding	✓
		(*b*) Describe any methods used to examine subgroups and interactions	✓
		(*c*) Explain how missing data were addressed	✓
		(*d*) *Cohort study*—If applicable, explain how loss to follow-up was addressed	✓
		Case-control study—If applicable, explain how matching of cases and controls was addressed	
		Cross-sectional study—If applicable, describe analytical methods taking account of sampling strategy	✓
		(*e*) Describe any sensitivity analyses	✓

Continued on next page

For peer review only

For peer review only - http://bmjopen.bmj.com/site/about/guidelines.xhtml

**Results**			page 9-11
Participants	13*	(a) Report numbers of individuals at each stage of study—eg numbers potentially eligible, examined for eligibility, confirmed eligible, included in the study, completing follow-up, and analysed	✓
		(b) Give reasons for non-participation at each stage	✓
		(c) Consider use of a flow diagram	✓
Descriptive data	14*	(a) Give characteristics of study participants (eg demographic, clinical, social) and information on exposures and potential confounders	✓
		(b) Indicate number of participants with missing data for each variable of interest	✓
		(c) *Cohort study*—Summarise follow-up time (eg, average and total amount)	✓
Outcome data	15*	*Cohort study*—Report numbers of outcome events or summary measures over time	✓
		Case-control study—Report numbers in each exposure category, or summary measures of exposure	
		Cross-sectional study—Report numbers of outcome events or summary measures	✓
Main results	16	(*a*) Give unadjusted estimates and, if applicable, confounder-adjusted estimates and their precision (eg, 95% confidence interval). Make clear which confounders were adjusted for and why they were included	✓
		(*b*) Report category boundaries when continuous variables were categorized	✓
		(*c*) If relevant, consider translating estimates of relative risk into absolute risk for a meaningful time period	
Other analyses	17	Report other analyses done—eg analyses of subgroups and interactions, and sensitivity analyses	✓
**Discussion**			page 11-15
Key results	18	Summarise key results with reference to study objectives	✓
Limitations	19	Discuss limitations of the study, taking into account sources of potential bias or imprecision. Discuss both direction and magnitude of any potential bias	✓
Interpretation	20	Give a cautious overall interpretation of results considering objectives, limitations, multiplicity of analyses, results from similar studies, and other relevant evidence	✓
Generalisability	21	Discuss the generalisability (external validity) of the study results	✓
**Other information**			page 16
Funding	22	Give the source of funding and the role of the funders for the present study and, if applicable, for the original study on which the present article is based	✓

*Give information separately for cases and controls in case-control studies and, if applicable, for exposed and unexposed groups in cohort and cross-sectional studies.

Note: An Explanation and Elaboration article discusses each checklist item and gives methodological background and published examples of transparent reporting. The STROBE checklist is best used in conjunction with this article (freely available on the Web sites of PLoS Medicine at http://www.plosmedicine.org/, Annals of Internal Medicine at http://www.annals.org/, and Epidemiology at http://www.epidem.com/). Information on the STROBE Initiative is available at www.strobe-statement.org.

For peer review only - http://bmjopen.bmj.com/site/about/guidelines.xhtml

BMJ Open

Risk of metabolic disorders in childless men - A population-based cohort study

Journal:	*BMJ Open*
Manuscript ID	bmjopen-2017-020293.R1
Article Type:	Research
Date Submitted by the Author:	17-May-2018
Complete List of Authors:	bungum, ane; Bispebjerg Hospital, department of occupational and environmental health<br>Glazer, Clara; Bispebjerg Hospital, department of occupational and environmental health<br>Bonde, Jens Peter; Copenhagen University Hospital Bispebjerg, Department of Occupational and Environmental Medicine<br>Nilsson, Peter; University of Lund, Clinical Sciences<br>Giwercman, Aleksander; Lund University, Department of Translational Medicine<br>Søgaard Tøttenborg, Sandra; Bispebjerg Hospital, department of occupational and environmental health
**Primary Subject Heading**:	Reproductive medicine
Secondary Subject Heading:	Epidemiology
Keywords:	Childlessness, Infertility, Metabolic syndrome, Diabetes, Register-based cohort study

SCHOLARONE™
Manuscripts

Title: Risk of metabolic disorders in childless men - A population-based cohort study

Authors: Ane Berger Bungum[1,3*], Clara Helene Glazer[1], Jens Peter Bonde[1], Peter M. Nilsson[2], Aleksander Giwercman[3], Sandra Søgaard Tøttenborg[1]

[1] Department of Occupational and Environmental Medicine, Bispebjerg University Hospital, DK-2400 Copenhagen NV, Denmark, [2] Department of Clinical Sciences Malmö, Lund University, Skåne University Hospital, S-20502 Malmö, Sweden, [3] Molecular Reproductive Medicine, Department of Translational Medicine, Lund University, S-21428 Malmö, Sweden

Correspondence and requests for reprints:

*Ane Berger Bungum**

Department of Occupational and Environmental Medicine

Capital Region of Denmark

Bispebjerg - Frederiksberg Hospital

Bispebjerg Bakke 23, Building 20C, 1. sal

DK-2400 Copenhagen NV

Denmark

Telephone: +45 30251797

E-mail: ane.berger.bungum@regionh.dk

For peer review only - http://bmjopen.bmj.com/site/about/guidelines.xhtml

Abstract

Objective: To study whether male childlessness is associated with an increased risk of metabolic disorders such as metabolic syndrome (MetS) and diabetes.

Design: A population-based cohort study

Setting: Not applicable.

Participants: 2 572 men from the population-based Malmö Diet and Cancer Cardiovascular Cohort (MDC-CC).

Interventions: None.

Main outcome measure(s): From cross-sectional analyses main outcome measures were odds ratios (OR) and 95% confidence intervals (CI) for MetS and diabetes among childless men. In prospective analyses Hazard ratios (HRs) and 95% CI for diabetes among childless men.

Results: At baseline, in males with a mean age of 57 years, the prevalence of MetS was 26% and 22% among childless men and fathers, respectively. Similarly we observed a higher prevalence of diabetes of 11% among childless men compared to 5% among fathers. In the cross-sectional adjusted analyses childless men had a higher risk of MetS and diabetes, with ORs of 1.22 (95% CI: 0.87;1.72) and 2.12 (95% CI: 1.34;3.36) compared to fathers. In the prospective analysis, during a mean follow-up of 18.3 years, we did not see any increase in diabetes risk among childless men (HR 1.02 [0.76;1.37]).

Conclusion(s): This study provides evidence of an association between male childlessness and a higher risk of MetS and diabetes. However, as these associations were found in cross-sectional analyses, reverse causation cannot be excluded.

For peer review only

Strengths and limitations of this study

- Malmö Diet and Cancer Cardiovascular Cohort (MDC-CC) is sampled from the background urban population, meaning that men from all socio-economic backgrounds were represented.
- This study has the longest mean follow-up among similar studies published.
- Childlessness was associated with increased risks of MetS and diabetes in cross-sectional analyses but not in prospective analyses. Using childlessness as a proxy of infertility may pose some challenges as the group of childless men is heterogeneous in relation to different causes of childlessness.
- We were unable to distinguish between fathers of biological or adopted children and fathers of children conceived after in vitro fertilisation (IVF).

For peer review only

Introduction

A man's reproductive health may not only reflect his chance to become a father, but may also be related to his general health [1]. In recent years, male childlessness and infertility have been reported to be associated with an increased risk of all-cause mortality and cardiovascular disease [2–5]. Male infertility has also been associated with a higher risk of metabolic disorders [3,6,7]. Metabolic syndrome (MetS) and type 2 diabetes are metabolic disorders, with increasing prevalence worldwide [8,9]. MetS is a syndrome consisting of a cluster of markers, including visceral obesity, hypertension, and hyperglycaemia [8]. Importantly, the syndrome may help to identify individuals at future risk of type 2 diabetes and cardiovascular disease [10–12].

Cross-sectional studies have demonstrated an association between factors related to male reproductive health (e.g. hypogonadism, reduced semen quality and erectile dysfunction) and MetS as well as type 2 diabetes [13–18], but whether poor reproductive health precedes MetS and diabetes or vice versa is uncertain due to the cross-sectional design of these studies. Two recent prospective studies found increased risk of developing diabetes among infertile men [3,7]. Authors of these prospective studies did not suggest a causal relationship for the association, but rather common aetiologies of infertility and diabetes such as shared genetics and factors related to endocrine regulation, lifestyle or *in-utero* exposures. However, these prospective studies failed to adjust for Body Mass Index (BMI), physical activity level, and other lifestyle factors considered as common risk factors for type 2 diabetes and poor male reproductive health [11,19]. Also, as type 2 diabetes can remain asymptomatic and undiagnosed for years, the measure of outcome of both prospective studies have a central limitation in common; dependence on health-seeking behaviour of the study participants.

For peer review only

Assessment of fertility status of a male is based on access to clinical and laboratory data, including semen analysis, which are difficult and costly to obtain in population-based studies. However, information regarding childlessness is more easily accessible and may be a feasible proxy for infertility. Therefore, using data from the Malmö Diet and Cancer Cardiovascular Cohort (MDC-CC), we aimed to examine whether male childlessness is associated with MetS and diabetes, while taking potential confounding lifestyle factors into account. We first examined the prevalence of MetS and diabetes in men with and without children, and next we assessed the incidence of diabetes during a mean follow-up of 18.3 years. Study participants were examined for diabetes both at baseline and follow-up clinical examination, limiting the influence of health seeking behaviour and giving reliable estimates of diabetes prevalence and incidence among childless Swedish men.

Materials and methods

Study population

The Malmö Diet and Cancer Cohort (MDC) is a population-based cohort of 30 446 Malmö residents born. During 1991 through 1996, 12 120 men (born between 1926 and 1945) and 18 326 women (born between 1923 and 1950) were enrolled [20]. The cohort had participation rate of 38% for men [21]. Following the initial acceptance letter, during the years of 1991-1994, half of the individuals in MDC randomly selected were invited to participate in a sub-cohort named MDC-CC. Of these, 2 572 men accepted the invitation (Figure 1).

Both at baseline (1991-1994) and follow-up (2007-2012) MDC-CC participants completed a questionnaire regarding marital status, number of children, and lifestyle factors (alcohol (g/day), smoking habits (regularly, occasionally, stopped, never), and total physical activity score (according to the Minnesota leisure time Physical activity questionnaire [22] - calculated as minutes/week for spring/summer/autumn/winter multiplied with an activity specific factor according to the type of

For peer review only

activity, e.g. running, walking)). Participants also underwent a clinical examination including body composition, blood pressure measurement, and collection of venous blood samples [21]. At follow-up examination where 1 522 men (59%) participated, the clinical examination also included an oral glucose tolerance test in study participants without known diabetes.

Ethical Approval

The MDC project was approved by the Ethics Committee of the Lund University (LU 51-90) and by the Swedish Data Inspection Agency.

Patient and public involvement

Patients and or public were not involved.

Information on childlessness

Information regarding childlessness came from two sources: the baseline questionnaire and the Swedish Tax Agency (STA). In the questionnaire, participants were asked *"Do you have any children?"* with reply options 'Yes' or 'No'. The STA holds the number of registered children and their respective birth dates. Data were linked using the unique 10-digit personal identification number assigned to all Swedish citizens. We linked these data sources to stratify the participants into four groups; 'Childless', 'One or more child', 'Conflicting information', and 'Unknown'. 'Conflicting information' appeared if a participant answered "No" to *"Do you have any children?"* in the baseline questionnaire, but was registered with one or more children in the STA. Men with 'Conflicting information' who became fathers after the entry date into the MDC-CC cohort were treated as 'Childless' as they were childless at baseline. The remaining men with 'Conflicting information' were registered as fathers in the registry of STA, and were therefore treated as having

For peer review only

'One or more child'. We used the designation "Unknown" if no information regarding children was available from the STA and if no answer was provided to *"Do you have any children?"* in the baseline questionnaire.

Assessment of MetS

MetS was defined according to the harmonized criteria of the International Diabetes Federation Task Force on Epidemiology and Prevention; National Heart, Lung, and Blood Institute; American Heart Association; World Heart Federation; International Atherosclerosis Society; and International Association for the Study of Obesity [23]. Accordingly, MetS was present if three or more of the following criteria were met: Fasting blood glucose (fB-glucose) level above 5.6 mmol/L or the use of anti-diabetic drugs, High Density Lipoprotein (HDL) cholesterol level below 1.03 mmol/L or the use of lipid modifying treatment, triglycerides level above 1.7 mmol/L or the use of lipid-lowering drugs, waist circumference higher than 102 cm and blood pressure above 130/85 mmHg, or the use of antihypertensive medicine [23]. Data regarding MetS criteria were only available from the baseline clinical examination.

Diagnosis of diabetes

The diabetes cases and the date of diagnosis were identified from 14 different data sources, including the Swedish National Diabetes register (NDR), The Swedish Prescribed Drug Register, and from baseline and follow-up screenings in MDC, MDC-CC, and the Malmö Preventive Project (MPP) [24]. These sources were used to identify prevalent cases of diabetes (type 1 and type 2) at baseline, and new-onset incident cases of diabetes (type 1 and type 2) during the follow-up period. In brief, individuals with a date of diagnosis registered in the NDR and/or Diabetes 2000 Register were considered to have diabetes. The same was true for individuals in the local HbA_{1c} Register in

For peer review only

Malmö with at least two $HbA_{1c} \geq 6\%$, those with the Tenth Edition of International Classification of Diseases (ICD10) codes E10-E14 and O244-O249, and corresponding ICD7-9 codes in the National Hospitalisation Register or in the Cause-of-death Register, and men in The Swedish Prescribed Drug Register with Anatomical Therapeutic Chemical (ATC) code A10.

From baseline questionnaires of MDC and MPP, participants who answered "Yes" to *"Do you have diabetes?"* and/or listed antidiabetic drugs were considered as patients with diabetes. At the baseline examination of MDC-CC, individuals were considered having diabetes if the fasting blood glucose (fB-glucose) measurement was ≥ 6.5 mmol/L. In MPP and at the MDC-CC follow-up, fB-glucose ≥ 6.5 mmol/L had to be verified through OGTT and or fP-glucose measurements. A full list of the diabetes diagnostic sources is provided in supplementary Table I. The participants were considered as having diabetes from their first diagnosis of diabetes while any and subsequent contradictory information about diabetes was ignored [25].

Statistical analyses

Cross-sectional analysis

To assess the association between male childlessness and metabolic disorders, the prevalence of MetS and diabetes at baseline 1991-1996 was compared among men with and without children by means of logistic regression and reported as odds ratios (OR) with corresponding 95% confidence intervals (CI). As childlessness could be a function of not having a partner, sensitivity analyses including only married men were also performed.

Prospective analysis

To assess whether the possible association between male childlessness and diabetes persisted or attenuated later in life, hazard ratios (HR) with 95% CIs of diabetes among childless men compared

For peer review only

to fathers were computed using Cox proportional hazard models. Men with pre-existing diabetes at baseline were excluded. The men were followed from enrolment into the MDC-CC until date of diabetes diagnosis, emigration, death, or end of follow-up 31st of December 2014. Kaplan-Meier plots allowed for visual evaluation of proportional hazards assumption. As with the cross-sectional analysis, we completed a sensitivity analysis including only married men.

Potential confounders were identified *a priori* using directed acyclic graphs [26]. Adjustments were performed in two steps in all analyses. The first adjustment step (*Model I*) included age in years, marital status (Married/Unmarried/Divorced/Widower), socio-economic index (SEI) (Workers, unskilled/Workers, skilled/Lower positioned official or salaried/Intermediate positioned official or salaried/Employers or self-employed), and educational attainment (No education/Primary school/Secondary school/High school/>One year education after high school/University degree). The second adjustment step (*Model II*) also included BMI in kg/m^2, alcohol consumption in grams per day, smoking habits (Regularly smoker/Occasional smoker/Stopped smoking/Never smoked) and physical activity level in minutes per week. The reason for the distinction between *Model I* and *Model II* is that it can be argued that BMI, alcohol consumption, smoking habits, and physical activity level mediates rather than confound the association between childlessness, MetS, and diabetes. For instance, smoking habits can affect a man's reproductive health, but on the contrary if a man becomes a father, this can affect his smoking habits.

In the statistical analyses, probability values (*p*-values) were not included, instead 95% CI for measures of association were reported to display the measure of precision [27]. All statistical tests were performed using SAS version 9.4.

Results

For peer review only

Among all 2 572 men, 422 had missing information regarding fatherhood status and were excluded from analyses. Twenty men had 'Conflicting information' regarding fatherhood, of which 18 men had registered children in the STA before baseline, and two men became fathers after baseline and thus treated as 'Childless'. Consequently, 2 150 men were included in the analyses of which 15% were childless and 85% fathers (Table I). The mean age in the cohort was 57 years at baseline. The baseline socio-demographic and lifestyle characteristics were equally distributed in general among childless men and fathers, except for the distribution of marital status, with 81% of fathers being married compared to only 37% of childless men being married.

Association between childlessness, MetS, and diabetes

MetS

The prevalence of MetS at baseline was 26% among childless men and 22% among fathers. . The major contributing factor for MetS among childless men was hyperglycaemia (Table II). The fully adjusted analyses (*Model II*) indicated an increased risk of MetS in childless men compared to fathers, (OR 1.22 [95% CI: 0.87;1.72]) (Table III). When comparing married childless men to married fathers the association became stronger and statistically significant with OR 1.62 [95% CI: 1.01; 2.60] in the fully adjusted model (Table III).

Diabetes

The prevalence of diabetes at baseline, was 11% among childless men and 5% among fathers. In the fully adjusted analysis (*Model II*) childless men had a higher risk of diabetes compared to fathers with OR 2.12 [95% CI: 1.34;3.36] (Table IV). The association persisted when comparing married fathers to married childless men (Table IV).

For peer review only

Risk of developing diabetes

The occurrence of new cases of diabetes was 20% among childless men and 22% among fathers. The mean follow up time was 18.3 years. The fully adjusted analysis (Model II) showed no increased risk of diabetes in childless men compared to fathers (Table V). However, the sensitivity analysis including only married men suggested an increased risk of diabetes among childless men compared to fathers (HR 1.13 [95% CI: 0.74;1.73]) (Table V).

Discussion

Main findings

Our study demonstrates cross-sectional associations between male childlessness, MetS, and diabetes. As expected, associations were generally stronger in analyses restricted to married men where a lack of reproductive opportunities can have been accounted for. The increased risk of MetS and diabetes among childless men could not be attributed to differences in lifestyle or socio-demographic characteristics between childless men and fathers.

Prior literature

Our findings are comparable to other cross-sectional studies reporting higher rates of medical co-morbidities, poorer general health status, and type 2 diabetes among infertile men [15,28–30]. However, whether infertility comes before diabetes or MetS and vice versa is still unclear. Some studies suggest hyperglycaemia which is a central element in both diabetes and MetS to affect the endocrine control of male reproductive function, and to impair spermatogenesis, sperm maturation, erectile function and ejaculation [11,13,31], and this makes revers causation of our study plausible. But results regarding the impact of MetS and diabetes on semen quality are conflicting [13,32]. Furthermore, our cross-sectional findings are also consistent with reports from the U.S. [3] and Denmark [7], that show male factor infertility to be associated with a 30-45% higher risk of

For peer review only

diabetes in prospective analyses, where the chance of reverse causation is highly limited, as exposure precedes outcome. Findings of these prospective studies are also supported by a recent Danish study [33] which found higher hospitalization rates for diabetes among men with poor semen quality and the authors of these latter mentioned studies suggested common aetiologies for male infertility, poor semen quality and diabetes. Our prospective results which showed no additional increase in risk can seem contradictory, but the mean age of the study population was more than 20 years higher than in the mentioned studies and this makes detection of early onset of diabetes difficult.

Mechanisms

The relationship between male reproductive health and somatic health is rather complex and different causal mechanisms and common aetiologies have been proposed for the association, namely shared genetic origins, *in utero*-, hormonal- or environmental/lifestyle factors [1–3,7,33]. More than 150 genes are linked to both male infertility and simultaneously involved in pathways important for several diseases, such as cancer, cardiovascular, and metabolic disorders [34]. Also, as male germ cell differentiation includes expression of up to 4% of all mammalian genes, it seems plausible that mutations in these genes could cause or contribute both to infertility, MetS as well as diabetes [35]. Further, as our cross-sectional results display a strong increase in risk of diabetes until the mean age of 57 years, and no additional increase in diabetes risk hereafter, as seen in our prospective results, this points to early onset of diabetes and support the hypothesis of genetic origins, as genetic predisposition increases the risk of early onset type 2 diabetes [36]. In addition to genetic defects, maternal health behaviour and environmental exposures during pregnancy have been hypothesized to act directly or through epigenetic mechanisms on the foetus and thereby influence both male reproductive health and somatic health [2,37].

For peer review only

Low testosterone levels have also been associated with infertility and an increased risk of MetS and diabetes, as testosterone plays an import role in glucose and lipid metabolism [38–40]. One study found low testosterone to predict development of MetS and onset of diabetes, even after adjusting for BMI, insulin resistance and other established risk factors for these conditions [39,41]. Another study among men with infertility problems and decreased sperm counts, reported low testosterone levels to be associated with higher levels of HbA_{1c} [42]. However, obesity, which is a strong risk factor for MetS and diabetes, is also known to lower testosterone levels, as testosterone is converted to oestradiol in adipose tissue. On the other hand, low testosterone levels are also known to increase obesity [43]. The causality of the relationship between low testosterone, MetS and diabetes, is therefore unclear but may be bidirectional [38].

Low socio-economic status and adverse lifestyle factors have been suspected to explain the association between poor reproductive health and somatic health. In the present study we adjusted for multiple lifestyle factors and socio-economic status, and associations were still seen. However, the operationalization of lifestyle factors may not sufficiently reduce the risk of confounding in the analysis of diabetes risk among childless men. This is due to the fact that information on lifestyle factors were only obtained at baseline and men with a diabetes diagnosis prior to baseline could have changed their lifestyle according to the disease. This makes it impossible to distinguish between pre-diagnostic lifestyle and post-diagnostic lifestyle, and only the lifestyle related to both exposure and outcome, namely the pre-diagnostic lifestyle, qualifies as a confounder. This may have induced some residual confounding in the analysis of diabetes risk, but not in the analysis of MetS risk as the diagnosis was based on information collected at baseline. Furthermore, our prospective analyses, which did not display the same association as our cross-sectional analyses, could also be influenced by results of clinical examination at baseline, and a wish among childless

For peer review only

men to live a healthier life style. Data regarding MetS criteria were only available from the baseline clinical examination, why a prospective analysis of the association between childlessness and MetS could not be performed.

Strengths and limitations

The present study has several strengths. *Firstly,* the MDC-CC is sampled from the background urban population, meaning that men from all socio-economic backgrounds were represented. A previous study showed no difference in baseline socio-demographic characteristics compared to study participants in the MDC [21]. Also, among Swedish men born between 1935 and 1945 one in six remained childless [44], which is comparable with the proportion of childless men in our cohort. *Secondly*, the mean cohort age at baseline was already advanced, and childlessness at an advanced age strengthens the assumption of infertility. *Thirdly*, the valid and comprehensive national Swedish registers provide information on emigrations, death, and disease limited loss to follow-up and made long-term follow up of more than 18 years in average possible – the longest mean follow-up among the few similar studies published to date [3,7,33].

Our study also has limitations. As for cross-sectional studies in general data regarding exposure and outcome were collected simultaneously, making it impossible to rule out reverse causation, However, as previously mentioned, studies have reported prospective associations between male infertility and risk of diabetes, in younger study populations than the present cohort [3,7,33], and the chance of reverse causation in prospective studies is low.

Furthermore, using childlessness as a proxy of infertility poses some challenges. The group of childless men is heterogeneous in relation to different causes of childlessness and do not only reflect male infertility (e.g. voluntarily childlessness, homosexual men, men with infertile partners

For peer review only

or single men). As only one in four childless Swedish men reported childlessness to be volitional [44], this limits the risk of our sample to reflect voluntary childlessness.

Also, we were unable to distinguish between fathers of biological or adopted children and fathers of children conceived after in vitro fertilisation (IVF). However, as adoption rates in Sweden were rather low at the time of baseline [45], misclassification would attenuate the estimate towards the null. Likewise, the chance of having children conceived after IVF was insignificant as assisted reproduction as treatment of impaired male fertility was not widely performed before and at the time of baseline in 1991-1994 [46]. This strengthens the usefulness of childlessness as a proxy for infertility in the present cohort.

Misclassifications can bias the estimated associations and the question is, whether men with MetS and diabetes are systematically more likely to be classified as childless. For instance if men with low socio-economic status are systematically more likely childless due to being single, and at higher risk of disease, this could lead to an overestimation of the association between infertility and MetS/Diabetes. However, by adjusting for socioeconomic status, and associated lifestyle factors as well as by confirming our findings in sensitivity analyses based on married men only this source of bias was minimized.

Unfortunately comparison of male participants versus male non-participants in either the MDC or the MDC-CC have not been done, but a study from 2001 concluded that mortality for men and women combined was higher in non-participants than in participants, which could reflect healthy selection bias in the cohort [21]. Selection bias occurs when conditioning on a common effect of exposure and outcome – in this case conditioning on survival of participants. In our analyses,

For peer review only

selection bias could occur if fatherhood status, and MetS or diabetes is directly or indirectly related to preterm death before initial enrolment into the cohort. For example if childless men are more likely to have lower socio-economic status (which itself is associated with increased risk of preterm death), and if men with MetS or diabetes are more likely to have comorbidities (which is related to preterm death).

Conclusion

In conclusion, our study showed a higher risk of MetS and diabetes among childless middle-aged men that could not be explained by differences in lifestyle, socio-demographic characteristics, or health seeking behaviour. This may support the hypothesis that a man's reproductive health is closely intertwined with his somatic health, however due to the nature of the cross-sectional design, where information on exposure and outcome is collected simultaneously, reverse causation cannot be excluded. While using childlessness as a proxy of male infertility may cause misclassification bias, it may still provide insight into a man's risk of disease. The simple objective measure of exposure enables for future studies to examine the association between male reproductive health and somatic health in large population-based cohorts.

Funding

This study was funded by ReproUnion and also supported by the Medical Research Council of Sweden (grant K2011-65X-20752-04-6), the Region Skåne County Council, the Ernhold Lundstrom Foundation, for the MDC-CC follow-up clinical examination.

Conflict of interest

None declared.

For peer review only

Author's contribution

J.P.B. and A.G. acquired funding for the study. A.B.B., C.H.G., S.S.T., A.G. and J.P.B. designed the study. A.B.B, S.S.T and C.H.G. analyzed data and A.B.B. wrote the manuscript. P.N. contributed with acquisition of data, critical discussion and revision of the paper. All authors contributed to data analysis/interpretation, critical revision of the paper and final approval of the manuscript.

Data sharing statement

No additional unpublished data.

For peer review only

References

1 Glazer CH, Bonde JP, Eisenberg ML, *et al.* Male Infertility and Risk of Nonmalignant Chronic Diseases: A Systematic Review of the Epidemiological Evidence. *Semin Reprod Med* 2017;**35**:282–90. doi:10.1055/s-0037-1603568

2 Jensen TK, Jacobsen R, Christensen K, *et al.* Good Semen Quality and Life Expectancy: A Cohort Study of 43,277 Men. *Am J Epidemiol* 2009;**170**:559–65. doi:10.1093/aje/kwp168

3 Eisenberg ML, Li S, Cullen MR, *et al.* Increased risk of incident chronic medical conditions in infertile men: analysis of United States claims data. *Fertil Steril* 2016;**105**:629–36. doi:10.1016/j.fertnstert.2015.11.011

4 Eisenberg ML, Park Y, Hollenbeck AR, *et al.* Fatherhood and the risk of cardiovascular mortality in the NIH-AARP Diet and Health Study. *Hum Reprod* 2011;**26**:3479–85. doi:10.1093/humrep/der305

5 Ringbäck Weitoft G, Burström B, Rosén M. Premature mortality among lone fathers and childless men. *Soc Sci Med* 2004;**59**:1449–59. doi:10.1016/j.socscimed.2004.01.026

6 Ventimiglia E, Capogrosso P, Serino A, *et al.* Metabolic syndrome in White-European men presenting for secondary couple's infertility: an investigation of the clinical and reproductive burden. *Asian J Androl* 2016;**19**:368–73. doi:10.4103/1008-682X.175783

7 Glazer CH, Bonde JP, Giwercman A, *et al.* Risk of diabetes according to male factor infertility: A register-based cohort study. *Hum Reprod* 2017;:[Epub ahead of print]. doi:10.1093/humrep/dex097

8 Alberti KGM, Zimmet P, Shaw J. The metabolic syndrome—a new worldwide definition. Lancet. 2005;**366**:1059–62. doi:10.1016/S0140-6736(05)67402-8

9 World Health Organization (WHO). Global report on diabetes. 2016.

10 Vliet-Ostaptchouk JV van, Nuotio M-L, Slagter SN, *et al.* The prevalence of metabolic syndrome and metabolically healthy obesity in Europe: a collaborative analysis of ten large cohort studies. *BMC Endocr Disord* 2014;**14**. doi:10.1186/1472-6823-14-9.

11 Kasturi SS, Tannir J, Brannigan RE. The Metabolic Syndrome and Male Infertility. *J Androl* 2008;**29**:251–9. doi:10.2164/jandrol.107.003731

12 Okoloise M. The metabolic syndrome as predictor of type 2 diabetes: The San Antonio Heart Study. *Diabetes Care* 2003;**26**:3153–9.

13 Ventimiglia E, Capogrosso P, Colicchia M, *et al.* Metabolic syndrome in white European

For peer review only

men presenting for primary couple's infertility: investigation of the clinical and reproductive burden. *Andrology* 2016;**4**:944–51. doi:10.1111/andr.12232

14 Delfino M, Imbrogno N, Elia J, *et al.* Prevalence of diabetes mellitus in male partners of infertile couples. *Minerva Urol Nefrol* 2007;**59**:131–5.

15 Bener A, Al-Ansari AA, Zirie M, *et al.* Is male fertility associated with type 2 diabetes mellitus? *Int Urol Nephrol* 2009;**41**:777–84. doi:10.1007/s11255-009-9565-6

16 Giugliano F, Maiorino M, Bellastella G, *et al.* Determinants of erectile dysfunction in type 2 diabetes. *Int J Impot Res* 2010;**22**:204–9. doi:10.1038/ijir.2010.1

17 Dunsmuir WD, Holmes SAV. The Aetiology and Management of Erectile, Ejaculatory, and Fertility Problems in Men with Diabetes Mellitus. *Diabet Med* 1996;**13**:700–8. doi:10.1002/(SICI)1096-9136(199608)13:8<700::AID-DIA174>3.0.CO;2-8

18 Laaksonen DE, Niskanen L, Punnonen K, *et al.* Sex hormones, inflammation and the metabolic syndrome: a population-based study. *Eur J Endocrinol* 2003;**149**:601–8. doi:10.1530/EJE.0.1490601

19 Jensen T, Anderson A, Jorgensen N, *et al.* Body mass index in relation to semen quality and reproductive hormonesamong 1,558 Danish men. *Fertil Steril* 2004;**82**:863–70. doi:10.1016/j.fertnstert.2004.03.056

20 Dahlin A, Nilsson P. Malmö Diet and Cancer Study Diabetes Endpoints. 2016.

21 Manjer J, Carlsson S, Elmståhl S, *et al.* The Malmö diet and cancer study: representativity, cancer incidence and mortality in participants and non-participants. *Eur J Cancer Prev* 2001;**10**:489–99.

22 Taylor HL, Jacobs DR, Schucker B, *et al.* A questionnaire for the assessment of leisure time physical activities. *J Chronic Dis* 1978;**31**:741–55. doi:10.1016/0021-9681(78)90058-9

23 Alberti KGM, Eckel RH, Grundy SM, *et al.* Harmonizing the Metabolic Syndrome. *Circulation* 2009;**120**:1640–5.

24 Magnusson M, Wang TJ, Clish C, *et al.* Dimethylglycine Deficiency and the Development of Diabetes. *Diabetes* 2015;**64**:3010–6. doi:10.2337/db14-1863

25 Borné Y, Smith JG, Nilsson PM, *et al.* Total and Differential Leukocyte Counts in Relation to Incidence of Diabetes Mellitus: A Prospective Population-Based Cohort Study. *PLoS One* 2016;**11**:e0148963. doi:10.1371/journal.pone.0148963

26 Greenland S, Pearl J, Robins JM. Causal Diagrams for Epidemiologic Research. *Epidemiology* 1999;**10**:37–48.

For peer review only

27 Rigby AS. Getting past the statistical referee: moving away from P-values and towards interval estimation. *Health Educ Res* 1999;**14**:713–5. doi:10.1093/her/14.6.713

28 Ventimiglia E, Capogrosso P, Boeri L, *et al.* Infertility as a proxy of general male health: results of a cross-sectional survey. *Fertil Steril* 2015;**104**:48–55. doi:10.1016/j.fertnstert.2015.04.020

29 Salonia A, Matloob R, Gallina A, *et al.* Are Infertile Men Less Healthy than Fertile Men? Results of a Prospective Case-Control Survey. *Eur Urol* 2009;**56**:1025–32. doi:10.1016/j.eururo.2009.03.001

30 Eisenberg ML, Li S, Behr B, *et al.* Relationship between semen production and medical comorbidity. *Fertil Steril* 2015;**103**:66–71. doi:10.1016/j.fertnstert.2014.10.017

31 Demir T, Demir O, Kefi A, *et al.* Prevalence of erectile dysfunction in patients with metabolic syndrome. *Int J Urol* 2006;**13**:385–8. doi:10.1111/j.1442-2042.2006.01310.x

32 Vignera S La, Condorelli R, Vicari E, *et al.* Diabetes Mellitus and Sperm Parameters. *J Androl* 2012;**33**:145–53. doi:10.2164/jandrol.111.013193

33 Latif T, Kold Jensen T, Mehlsen J, *et al.* Semen quality is a predictor of subsequent morbidity. A Danish cohort study of 4,712 men with long-term follow-up. *Am J Epidemiol* 2017;:[Epub ahead of print]. doi:10.1093/aje/kwx067

34 Tarín JJ, García-Pérez MA, Hamatani T, *et al.* Infertility etiologies are genetically and clinically linked with other diseases in single meta-diseases. *Reprod Biol Endocrinol* 2015;**13**. doi:10.1186/s12958-015-0029-9

35 Matzuk MM, Lamb DJ. The biology of infertility: research advances and clinical challenges. *Nat Med* 2008;**14**:1197–213. doi:10.1038/nm.f.1895

36 Wilmot E, Idris I. Early onset type 2 diabetes: risk factors, clinical impact and management. *Ther Adv Chronic Dis* 2014;**5**:234–44. doi:10.1177/2040622314548679

37 Skakkebaek NE, Rajpert-De Meyts E, Buck Louis GM, *et al.* Male Reproductive Disorders and Fertility Trends: Influences of Environment and Genetic Susceptibility. *Physiol Rev* 2016;**96**:55–97.

38 Kelly DM, Jones TH. Testosterone: A metabolic hormone in health and disease. *J Endocrinol* 2013;**217**:R25-45. doi:10.1530/JOE-12-0455

39 Laaksonen DE, Niskanen L, Punnonen K, *et al.* Testosterone and sex hormone-binding globulin predict the metabolic syndrome and diabetes in middle-aged men. *Diabetes Care* 2004;**27**:1036–41. doi:10.2337/diacare.27.5.1036

For peer review only

40 Brand JS, Rovers MM, Yeap BB, *et al.* Testosterone, sex hormone-binding globulin and the metabolic syndrome in men: An individual participant data meta-analysis of observational studies. *PLoS One* 2014;**9**:e100409. doi:10.1371/journal.pone.0100409

41 Li C, Ford ES, Li B, *et al.* Association of Testosterone and Sex Hormone-Binding Globulin With Metabolic Syndrome and Insulin Resistance in Men. *Diabetes Care* 2010;**33**:1618–24. doi:10.2337/dc09-1788

42 Bobjer J, Bogefors K, Isaksson S, *et al.* High prevalence of hypogonadism and associated impaired metabolic and bone mineral status in subfertile men. *Clin Endocrinol (Oxf)* 2016;**85**:189–95. doi:10.1111/cen.13038

43 Kelly DM, Jones TH. Testosterone and obesity. *Obes Rev* 2015;**16**:581–606. doi:10.1111/obr.12282

44 Statistics Sweden (SCB). Childbearing patterns of different generations. 2011.

45 Statistics Sweden (SCB). Internationella adoptioner minskar. 2013.

46 Gottvall K, Lundqvist E, Källén K. Graviditeter, förlossningar och nyfödda barn. Medicinska födelseregistret 1973-2014. Assisterad befruktning 1991-2013. 2015.

For peer review only

Table I: Socio-demographic and lifestyle characteristics of men with and without children at baseline. Means (SD) and proportions

	**Childless men (*n*=333)**	**Fathers (*n*=1 817)**
**Age (years)** (*n*=2 150)	57.3 (6.1)	57.1 (5.9)
**Marital status** (*n*=2 052)		
- **Married (%)**	37	81
- **Unmarried (%)**	51	3
- **Divorced (%)**	8	14
- **Widower (%)**	4	2
**Socio-economic index** (*n*=1 927)		
- **Employers and self-employed (%)**	16	22
- **Official/Salaried, intermediate position (%)**	21	24
- **Official/Salaried, lower position (%)**	21	15
- **Workers, skilled (%)**	17	21
- **Workers, unskilled (%)**	25	18
**Highest level of education** (*n*=2 056)		
- **No education (%)**	0	1
- **Primary school (%)**	50	45
- **Secondary school (%)**	22	20
- **High school (%)**	11	12

For peer review only

- **>1 year education after high school (%)**	7	10
- **University degree (%)**	10	12
**BMI (kg/m^2)** (*n*=2 148)	26 (4.1)	26.1 (3.3)
**Alcohol (g/day)** (*n*=2 057)	14.6 (18.4)	15.8 (15.2)
**Present smoker** (*n*=2 058)		
- **Regularly (%)**	24	22
- **Occasionally (%)**	4	5
- **Stopped (%)**	38	43
- **Never (%)**	34	30
**Physical activity score†** (*n*=2 041)	8 675.3 (6 771.0)	8 361.9 (6 224.9)

*SD (standard deviation)

† Minutes/week for spring/summer/autumn/winter multiplied with an activity specific factor according to the type of activity, e.g. running, walking.

For peer review only

Table II: Odds ratio (OR) with 95% confidence intervals (95% CI) of metabolic syndrome (MetS) components in childless men (*n*=333) compared to fathers (*n*=1 817) at baseline

	**No. of cases among childless men (vs. fathers)**	**OR [95% CI]**
**Hyperglycaemia ***	88 (335)	1.59 [1.21;2.08]
**Hypo-HDL cholesterolemia †**	102 (670)	0.78 [0.60;1.01]
**Hyperlipidemia ‡**	97 (515)	1.04 [0.80;1.34]
**Waist circumference >102 cm**	54 (270)	1.11 [0.81;1.53]
**Hypertension §**	222 (1160)	1.13 [0.88;1.45]

* Hyperglycemia defined as a fasting blood glucose level ≥ 5.6 mmol/L or by the use of anti-diabetic medicine

† Hypo-HDL cholesterolemia defined as HDL<1.03 mmol/L or by the use of drug treatment

‡ Hyperlipidemia defined as Triglycerides≥1.7 mmol/L or by the use of lipid lowering drugs

§ Hypertension (elevated blood pressure) defined by ≥130/85 mmHg or by the use of antihypertensive drugs

For peer review only

Table III: Odds ratio (OR) with 95% confidence intervals (95% CI) for MetS in childless men relative to fathers

**Total population (*n*=2 150)**				
		**Crude**	**Model I ***	**Model II †**
	***n* cases**	**OR [95% CI]**	**OR [95% CI]**	**OR [95% CI]**
**Fathers (*n*=1 817)**	402 (22.1%)	1 (ref)	1 (ref)	1 (ref)
**Childless men (*n*=333)**	85 (25.5%)	1.21 [0.92;1.58]	1.22 [0.92;1.64]	1.22 [0.87;1.72]
**Only married men (*n*=1 515)**				
		**Crude**	**Model I ‡**	**Model II §**
	***n* cases**	**OR [95% CI]**	**OR [95% CI]**	**OR [95% CI]**
**Fathers (*n*=1 392)**	317 (22.8%)	1 (ref)	1 (ref)	1 (ref)
**Childless men (*n*=123)**	42 (34.2%)	1.76 [1.19;2.61]	1.66 [1.11;2.49]	1.62 [1.01;2.60]

* Model 1: Adjusted for age, marital status, SEI and education (*n*=1 923)

† Model 2: Adjusted for age, marital status, SEI, education, BMI, alcohol (g/day), smoking and physical activity score (*n*=1 895)

‡ Model I: Adjusted for age, SEI and education (*n*=1 414)

§ Model II: Adjusted for age, SEI, education, BMI, alcohol (g/day), smoking and physical activity score (*n*=1 396)

Table IV: Odds ratio (OR) with 95% confidence intervals (95% CI) for diabetes in childless men relative to men with children

**Total population (*n*=2 150)**				
		**Crude**	**Model I ***	**Model II †**
	***n* cases**	**OR [95% CI]**	**OR [95% CI]**	**OR [95% CI]**

For peer review only

**Fathers (*n*=1 817)**	87 (4.8%)	1 (ref)	1 (ref)	1 (ref)
**Childless men (*n*=333)**	35 (10.5%)	2.34 [1.55;3.52]	2.26 [1.44;3.54]	2.12 [1.34;3.36]
**Only married men (*n*=1 515)**				
		**Crude**	**Model I ‡**	**Model II §**
	***n* cases**	**OR [95% CI]**	**OR [95% CI]**	**OR [95% CI]**
**Fathers (*n*=1 392)**	64 (4.6%)	1 (ref)	1 (ref)	1 (ref)
**Childless men (*n*=123)**	13 (10.6%)	2.45 [1.30;4.59]	2.29 [1.18;4.43]	2.05 [1.03;4.08]

* Model I: Adjusted for age, marital status, SEI and education (*n*=1 923)

† Model II: Adjusted for age, marital status, SEI, education, BMI, alcohol (g/day), smoking and physical activity score (*n*=1 895)

‡ Model I: Adjusted for age, SEI and education (*n*=1 414)

§ Model II: Adjusted for age, SEI, education, BMI, alcohol (g/day), smoking and physical activity score (*n*=1 396)

Table V: Hazard ratio (HR) with 95% confidence intervals (95% CI) for diabetes in childless men relative to fathers

**Total population (*n*=2 028)**				
		**Crude**	**Model I ***	**Model II †**
	***n* cases**	**HR [95% CI]**	**HR [95% CI]**	**HR [95% CI]**
**Fathers (*n*=1 730)**	373 (21.6%)	1 (ref)	1 (ref)	1 (ref)
**Childless men (*n*=298)**	60 (20.1%)	1.05 [0.80;1.38]	1.12 [0,85;1.49]	1.02 [0.76;1.37]
**Only married men (*n*=1 438)**				
		**Crude**	**Model I ‡**	**Model II §**

For peer review only

	n cases	HR [95% CI]	HR [95% CI]	HR [95% CI]
**Fathers (*n*=1 328)**	281 (21.2%)	1 (ref)	1 (ref)	1 (ref)
**Childless men (*n*=110)**	24 (21.8%)	1.17 [0.77;1.77]	1.20 [0,79;1.83]	1.13 [0.74;1.73]

* Model I: Adjusted for age, marital status, SEI, and education (*n*=1 820)

† Model II: Adjusted for age, marital status, SEI, education, BMI, alcohol (g/day), smoking, and physical activity score (*n*=1 794)

‡ Model I: Adjusted for age, SEI and education (*n*=1 346)

§ Model II: Adjusted for age, SEI, education, BMI, alcohol (g/day), smoking and physical activity score (*n*=1 330)

Figure legend (figure 1)

Malmö diet and cancer cardiovascular cohort (MDC-CC) recruitment

For peer review only

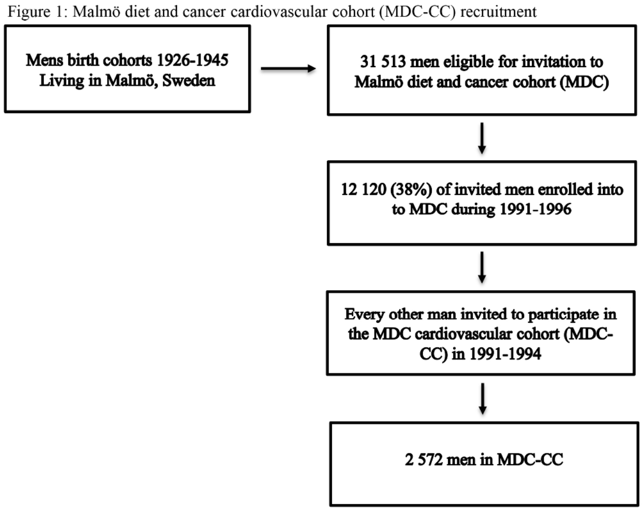

Figure 1: Malmö diet and cancer cardiovascular cohort (MDC-CC) recruitment

254x190mm (300 x 300 DPI)

Supplementary table I: Fourteen different sources of data were used to identify diabetic cases among men included in MDC-CC.

**Source of data**	**Diabetes criteria**
The Swedish National Diabetes Register (NDR)	All registered individuals
The Diabetes 2000 Registry	All registered individuals
The HbA1c register at Clinical Chemistry, Malmö	Individuals with at least two HbA1c ≥ 6% (not on the same day)
The Swedish Hospital Discharge Register (also The National Inpatient Register (IPR))	Individuals with the ICD10 codes E10-E14 and O244-O249 (corresponding ICD7-9 codes)
The Swedish National Patient Register – Outpatient Care	Individuals with the ICD10 codes E10-E14 and O244-O249 (corresponding ICD7-9 codes)
The Swedish Cause-of-death Register	Individuals with the ICD10 codes E10-E14 and O244-O249 (corresponding ICD7-9 codes)
The Swedish Prescribed Drug Register	Individuals with ATC code A10
MPP baseline screening (1974-92)	Based on questionnaire, fB-glucose ≥ 6.5 mmol/L and glucose ≥ 11 mmol/L at 120 minutes OGTT
MPP 6-year rescreening (1981-89)	Based on questionnaire, fB-glucose ≥ 6.5 mmol/L and glucose ≥ 11 mmol/L at 120 minutes OGTT
MPP rescreening (2002-06)	Based on questionnaire, list of antidiabetic drugs and fP-glucose ≥ 7 mmol/L
MDC baseline screening (1991-96)	Based on questionnaire and list of antidiabetic drugs
MDC cardiovascular cohort baseline screening (1992-94)	Based on fB-glucose ≥ 6.5 mmol/L
MDC 5-year rescreening (1997-2001)	Based on questionnaire and list of antidiabetic drugs

For peer review only

MDC cardiovascular cohort rescreening (2007-12)	Based on questionnaire, list of antidiabetic drugs, fB-glucose ≥ 6.5 mmol/L verified by fP-glucose ≥ 7 mmol/L and glucose ≥ 11 mmol/L at 120 minutes OGTT

For peer review only

STROBE Statement—checklist of items that should be included in reports of observational studies

	Item No	Recommendation	
**Title and abstract**	1	(*a*) Indicate the study's design with a commonly used term in the title or the abstract	✓ Page 1, line 1
		(*b*) Provide in the abstract an informative and balanced summary of what was done and what was found	✓ Page 2, line 9-21
**Introduction**			
Background/rationale	2	Explain the scientific background and rationale for the investigation being reported	✓ Page 5, line 2-24
Objectives	3	State specific objectives, including any prespecified hypotheses	✓ Page 6, line 5-13
**Methods**			
Study design	4	Present key elements of study design early in the paper	✓ Page 6, line 17-23
Setting	5	Describe the setting, locations, and relevant dates, including periods of recruitment, exposure, follow-up, and data collection	✓ Page 6, line 17-23 Page 7, line 1-10
Participants	6	(*a*) *Cohort study*—Give the eligibility criteria, and the sources and methods of selection of participants. Describe methods of follow-up<br>*Case-control study*—Give the eligibility criteria, and the sources and methods of case ascertainment and control selection. Give the rationale for the choice of cases and controls<br>*Cross-sectional study*—Give the eligibility criteria, and the sources and methods of selection of participants	✓ Page 6, line 17-23 Page 7, line 1-10
		(*b*) *Cohort study*—For matched studies, give matching criteria and number of exposed and unexposed<br>*Case-control study*—For matched studies, give matching criteria and the number of controls per case	✓ Page 7, line 16-24 Page 8, line 1-5
Variables	7	Clearly define all outcomes, exposures, predictors, potential confounders, and effect modifiers. Give diagnostic criteria, if applicable	✓ Page 7, line 16-24 Page 8, line 1-24 Page 9, line 1-24
Data sources/ measurement	8*	For each variable of interest, give sources of data and details of methods of assessment (measurement). Describe comparability of assessment methods if there is more than one group	✓ Page 10, line 1-22 Page 11, line 1-3
Bias	9	Describe any efforts to address potential sources of bias	✓ Page 10, line 9-22
Study size	10	Explain how the study size was arrived at	✓ Page 6, line 17-23
Quantitative variables	11	Explain how quantitative variables were handled in the analyses. If applicable, describe which groupings were chosen and why	
Statistical methods	12	(*a*) Describe all statistical methods, including those used to control for confounding	✓ Page 9, line 18-22 Page 10, line 1-22
		(*b*) Describe any methods used to examine subgroups and interactions	
		(*c*) Explain how missing data were addressed	
		(*d*) *Cohort study*—If applicable, explain how loss to follow-up was addressed<br>*Case-control study*—If applicable, explain how matching of cases and controls was addressed<br>*Cross-sectional study*—If applicable, describe analytical methods taking account of sampling strategy	✓ Page 10, line 4-5
		(*e*) Describe any sensitivity analyses	✓ Page 9, line 21-22 Page 10, line 6-7

Continued on next page

For peer review only

**Results**			
Participants	13*	(a) Report numbers of individuals at each stage of study—eg numbers potentially eligible, examined for eligibility, confirmed eligible, included in the study, completing follow-up, and analysed	✓ Page 11, line 6-10
		(b) Give reasons for non-participation at each stage	
		(c) Consider use of a flow diagram	✓ Figure I
Descriptive data	14*	(a) Give characteristics of study participants (eg demographic, clinical, social) and information on exposures and potential confounders	✓ Table I
		(b) Indicate number of participants with missing data for each variable of interest	✓ Table I
		(c) *Cohort study*—Summarise follow-up time (eg, average and total amount)	✓ Page 12, line 10
Outcome data	15*	*Cohort study*—Report numbers of outcome events or summary measures over time	✓ Page 11, line 17-24 Page 12, 2-17
		Case-control study—Report numbers in each exposure category, or summary measures of exposure	
		Cross-sectional study—Report numbers of outcome events or summary measures	
Main results	16	(*a*) Give unadjusted estimates and, if applicable, confounder-adjusted estimates and their precision (eg, 95% confidence interval). Make clear which confounders were adjusted for and why they were included	✓ Page 11, line 17-24 Page 12, 2-17 Table II-V
		(*b*) Report category boundaries when continuous variables were categorized	
		(*c*) If relevant, consider translating estimates of relative risk into absolute risk for a meaningful time period	
Other analyses	17	Report other analyses done—eg analyses of subgroups and interactions, and sensitivity analyses	✓ Table III-V
**Discussion**			
Key results	18	Summarise key results with reference to study objectives	✓ Page 12, line 19-23
Limitations	19	Discuss limitations of the study, taking into account sources of potential bias or imprecision. Discuss both direction and magnitude of any potential bias	✓ Page 16, line 15-24 Page 17, line 1-24 Page 18, line 1-24
Interpretation	20	Give a cautious overall interpretation of results considering objectives, limitations, multiplicity of analyses, results from similar studies, and other relevant evidence	✓ Page 19, line 16-25 Page 20, line 1-2
Generalisability	21	Discuss the generalisability (external validity) of the study results	✓ Page 17, line 1-24 Page 18, line 1-24
**Other information**			
Funding	22	Give the source of funding and the role of the funders for the present study and, if applicable, for the original study on which the present article is based	✓ Page 20, line 5-7

*Give information separately for cases and controls in case-control studies and, if applicable, for exposed and unexposed groups in cohort and cross-sectional studies.

Note: An Explanation and Elaboration article discusses each checklist item and gives methodological background and published examples of transparent reporting. The STROBE checklist is best used in conjunction with this article (freely available on the Web sites of PLoS Medicine at http://www.plosmedicine.org/, Annals of Internal Medicine at http://www.annals.org/, and Epidemiology at http://www.epidem.com/). Information on the STROBE Initiative is available at www.strobe-statement.org.

For peer review only - http://bmjopen.bmj.com/site/about/guidelines.xhtml
